# Early-life growth and cellular heterogeneity in the short-lived African turquoise killifish telencephalon

**DOI:** 10.1242/bio.061984

**Published:** 2025-04-22

**Authors:** Caroline Zandecki, Valerie Mariën, Rajagopal Ayana, Jolien Van houcke, Lutgarde Arckens, Eve Seuntjens

**Affiliations:** ^1^ Laboratory of Developmental Neurobiology, Animal Physiology and Neurobiology Section, Department of Biology, KU Leuven, 3000 Leuven, Belgium; ^2^ Laboratory of Neuroplasticity and Neuroproteomics, Animal Physiology and Neurobiology Section, Department of Biology, KU Leuven, 3000 Leuven, Belgium; ^3^ KU Leuven Brain Institute, 3000 Leuven, Belgium; ^4^ Leuven Institute for Single Cell Omics, 3000 Leuven, Belgium

**Keywords:** Teleost, Post-embryonic development, Telencephalon, Progenitor diversity, Excitatory and inhibitory neurons

## Abstract

The African turquoise killifish (*Nothobranchius furzeri*) is becoming a favorable model for neurobiological research. The combination of a short lifespan and a declining neuroregenerative capacity upon aging makes it ideally suited for research on brain aging and regeneration. A remarkable cellular diversity makes up the young-adult killifish telencephalon, characterized by highly proliferative non-glial progenitors and spatially distinct radial glia subtypes. In contrast to a relatively slow embryonic development, hatching is followed by a period of accelerated growth, in which the brain experiences a period of rapid expansion and maturation. In this study, we quantified the growth progression and maturation of the killifish telencephalon during early post-embryonic development. We discovered that, similar to in zebrafish, neuro-epithelial cells abut the neurogenic niches from early life onwards. Spatial data revealed qualitative and quantitative differences along the anterior-posterior axis and between pallial and subpallial regions in terms of growth pace. We confirmed generation of GABAergic neurons from the subpallial neurogenic niche and glutamatergic neurons from two pallial niches. Our data further showed a more widespread appearance of inhibitory neurons at hatching compared to in zebrafish.

## Teaser

**Summary:** This study charts spatial patterns of growth of the African turquoise killifish telencephalon from hatching to adulthood, and identifies the progenitors and neurons present during this phase of accelerated brain growth.

## INTRODUCTION

The African turquoise killifish (*Nothobranchius furzeri*) has developed into an established model for age-related research, in part because of its remarkably short natural lifespan. Annual *Nothobranchius* fishes inhabit temporary water ponds in Southeast Africa. The unpredictable and temporally restricted alternations of rainy and dry seasons pressured these fishes to develop explosive growth and attain fast sexual maturation (Valdesalici and Cellerino, 2003; Blažek et al., 2013; Reichard and Polačik, 2019). The GRZ *N. furzeri* strain has the shortest lifespan recorded and maintains this characteristic under laboratory conditions (Valdesalici and Cellerino, 2003; Cellerino et al., 2016; Van houcke et al., 2021).

Research on killifish has been carried out to study the impact of aging on various biological processes and systems, such as the immune system (Bradshaw et al., 2022), circadian rhythm (Barth et al., 2021), epigenetics (Zupkovitz et al., 2021), wound healing (Örling et al., 2023) and the central nervous system (Tozzini et al., 2012; Van houcke et al., 2021; Vanhunsel et al., 2021). This revealed that, in contrast to other highly regenerative species, the brain of this short-lived teleost loses its impressive regeneration capacity upon aging, and even adopts mammalian traits, such as glial scar formation upon injury (Wendler et al., 2015; Van houcke et al., 2021; Vanhunsel et al., 2022). These human-like aging features of the killifish central nervous system have prompted researchers to investigate the occurrence of spontaneous neurodegeneration and other age-related disease hallmarks for diseases like Parkinson's disease and amyotrophic lateral sclerosis (Valenzano et al., 2006; Matsui et al., 2019; Bagnoli et al., 2022; Louka et al., 2022; Bergmans et al., 2023; de Bakker and Valenzano, 2023).

The period of explosive growth after hatching is preceded by very slow embryonic development in the killifish. Depending on the time spent in diapause, embryonic development can take 3-4 weeks to multiple years (Genade et al., 2005; Blažek et al., 2013; Api et al., 2018). This is in stark contrast to the extremely fast development of common teleost model systems such as zebrafish (*Danio rerio*), goldfish (*Carassius auratus*) and medaka (*Oryzias latipes*), with an embryonic development period of 2, 3 and 9 days, respectively (Kimmel et al., 1995; Iwamatsu, 2004; Tsai et al., 2013). After fast embryonic development, zebrafish mature slowly into a sexually mature adult in approximately 3 months and do not experience a period of accelerated growth (Parichy et al., 2009). Killifish, on the contrary, reach sexual maturity in 5-6 weeks. The growth of the killifish pallial surface, for example, is reported to be three times faster than that of the zebrafish in the first 2 weeks of development (Coolen et al., 2020), but the growth pattern and appearance of progenitors and neural cell types from hatching to adult have not been documented yet.

The neurogenic niches and neural progenitor cells are identified for the adult killifish brain and comparisons could be made with established model systems like zebrafish and mouse. In the telencephalon, three neurogenic regions are identified, as was done for zebrafish (Adolf et al., 2006; Grandel et al., 2006; Tozzini et al., 2012). Region I is located in the subpallium, while region II stretches over the pallial surface, and region III is found at the posterior zone of the dorsal pallium (Tozzini et al., 2012). In zebrafish, radial glia (RG) are the main progenitor cell type and are responsible for the production of neurons both during development and in the adult brain (Furlan et al., 2017; Coolen et al., 2020), although heterogeneity in the progenitor population is also reported in zebrafish (Grandel et al., 2006; März et al., 2010). The progenitors responsible for the bulk of proliferation in the killifish telencephalon, termed non-glial progenitors (NGPs), lack the typical RG transcriptional profile yet possess an apical domain and basal process (Coolen et al., 2020; Ayana et al., 2024). NGPs are present already in the young post-hatching killifish brain, and, at 6 weeks, they can be recognized by expression of PCNA, MSH1, HMGB2A and STMN1A (Coolen et al., 2020; Ayana et al., 2024). Our previous single-cell transcriptomics and spatial analysis further revealed a tremendous progenitor cell diversity in the adult killifish telencephalon. Next to two NGP subtypes, we identified four RG subtypes and multiple intermediate cell states. The RG subclusters display an astroglial (Astro-RG1/Astro-RG2, marked by SLC1A2 and/or CX43, respectively), neuroepithelial (NE-RG3, marked by ZIC2) or ependymal (EPD-RG4, marked by EPD) transcriptional profile (Fig. S1), and all have distinct spatial locations in the adult telencephalon (Ayana et al., 2024).

Since previous killifish research has mainly focused on aging, unique adaptations necessary for accelerated growth in the early-life stages may have been overlooked. Besides the non-glial progenitors that have been discovered by Coolen et al. (2020) to be present in the brain at 2 weeks of age, it is unclear which of the other progenitor cell types found at 6 weeks are present early on. Second, it was unknown whether the explosive growth has a similar temporal mode of generating neurons in pallium and subpallium compared to zebrafish. In this study, we therefore investigated the growth progression of the killifish telencephalon during early post-embryonic development, quantified the pattern of growth and charted the cellular heterogeneity present during this explosive growth phase. We additionally reflected on the differences and similarities with zebrafish and other relevant model systems.

## RESULTS

### Explosive growth in the post-embryonic telencephalon

**Fig. 1.**
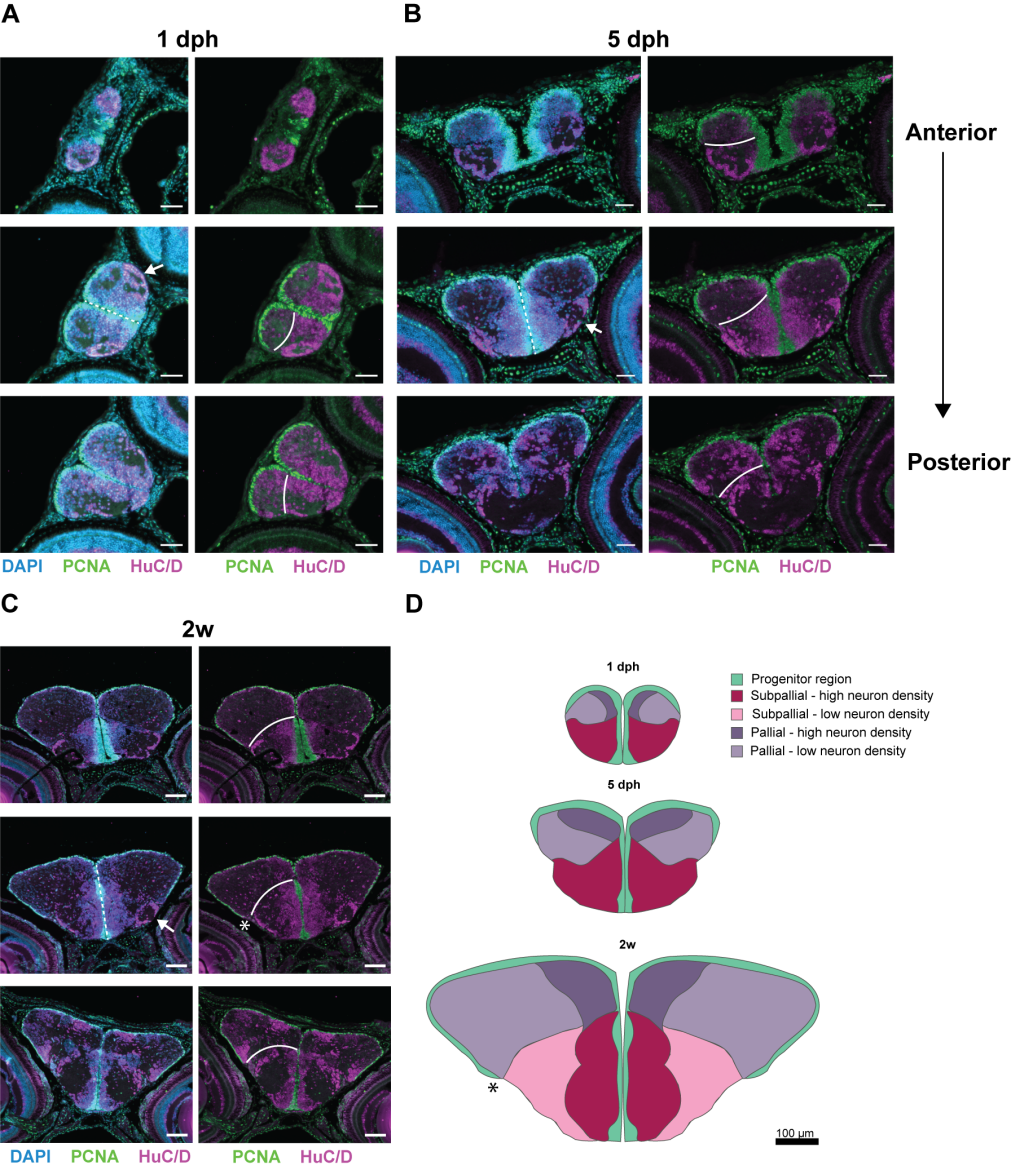
**Explosive growth of dorsal and ventral telencephalic domains.** (A-C) Immunohistochemical staining for PCNA (green) and HuC/D (magenta) in combination with a nuclear stain (DAPI, blue) in coronal brain sections. Three levels of the anterior-posterior axis – anterior-mid-posterior – are shown for killifish at 1 dph (A), 5 dph (B) and 2 weeks (C), from anterior to posterior. White lines represent the pallial-subpallial border. (D) On scale illustration summarizing the distribution of progenitor (green) and neuronal regions (shades of red/pink/purple) on coronal telencephalic sections of juveniles at 1 dph, 5 dph, and 2 weeks. Example sections of a comparable anterior-posterior level were selected based on the presence of a specific ventrolateral neuronal cluster (arrows, A-C) and the progenitor region extending from the dorsal to the ventral surface (dashed lines, A-C). (A-B) Scale bars: 50 µm (A,B); 100 µm (C,D). dph, days post-hatching; w, week.

To chart the source of post-hatching growth and morphogenesis of the telencephalon, we first visualized the proliferative progenitor and postmitotic neuronal domains at different stages from hatching to young-adult age (6 weeks). We used the proliferation marker PCNA to visualize cell division at 1 day post-hatching (dph), 5 dph, 2 weeks and 6 weeks, in combination with the pan-neuronal marker HuC/D (Elavl3/4) (Fig. 1A-C; Fig. S2A,B). At 1 dph and 5 dph, PCNA^+^ progenitors occupied one continuous region across the pallial surface from medial to lateral (Fig. 1A,B). The olfactory bulb was not directly covered with a progenitor layer at the dorsal side, visible in the rostral-most section at 1 dph (Fig. 1A). This aligns with what is known in the developing zebrafish, in which proliferating cells cover the dorsal surface between 2 and 5 days post-fertilization (dpf), but stay absent dorsally of the olfactory bulb (Folgueira et al., 2012). At 2 weeks, we detected that the PCNA^+^ pallial progenitor layer expanded further into the posterior pallial region (Fig. 1C,D, asterisk). At this stage, the overall structure resembles that of the adult telencephalon, yet it still undergoes a massive expansion from 2 weeks onwards. This could be observed when comparing the juvenile (2 weeks) telencephalon to the young-adult (6 weeks) telencephalon (Fig. S2A,B). To visualize the absolute difference in overall size of the telencephalon upon development, we created a representative illustration at the mid-anterior-posterior level of all ages discussed (Fig. 1D; Fig. S2B). Besides cell proliferation, the maturation of neuronal circuitry and growth of neuropil is a major driver of volumetric expansion of nervous tissue. To probe for neuropil expansion, we immunostained at different stages (5 dph, 2 weeks and 6 weeks) for the synaptic protein SV2, which reliably labels neuropil (Fig. S2D-F). We quantified the stained surface at different anterior-posterior levels and found significant increases between all time points and at all levels (Fig. S2C).

We further delineated the growth progression from hatchling to young-adult by measuring the horizontal and vertical expansion of the pallium and subpallium (Fig. S3). These absolute numbers indicate a greater expansion of the pallium compared to the subpallium in all directions measured. In the first week post-hatching, the dorsal telencephalon (Fig. 1D, purple, pallium), started expanding laterally and grew beyond the lateral subpallial edge (Fig. 1D, pink). The progenitor region covering the dorsal surface stretched along the expanding pallium. Stretching of the pallial surface in the first 2 weeks post-hatching was quantified by Coolen et al. (2020) and revealed that the dorsal surface increased 5-fold in this period. From 1 dph to 2 weeks, we measured a 4-fold increase, on average, in the pallium, confirming their results (Fig. S3). The subpallium, on the other hand, was proportionally bigger than the pallium at hatchling stages (1-5 dph) and was very dense in neurons compared to the pallium (Fig. 1A,B, the pallial-subpallial border is indicated with a white line). The surface of the proliferative zone of the subpallium did not stretch out as much as the pallium while the telencephalon increased in size. In general, we concluded that the pallial growth (10-fold increase) contributed most to the massive expansion of the telencephalon (overall 7-fold increase) from hatching to young-adult.

### Birth dating reveals two different stacking processes within the telencephalon

**Fig. 2.**
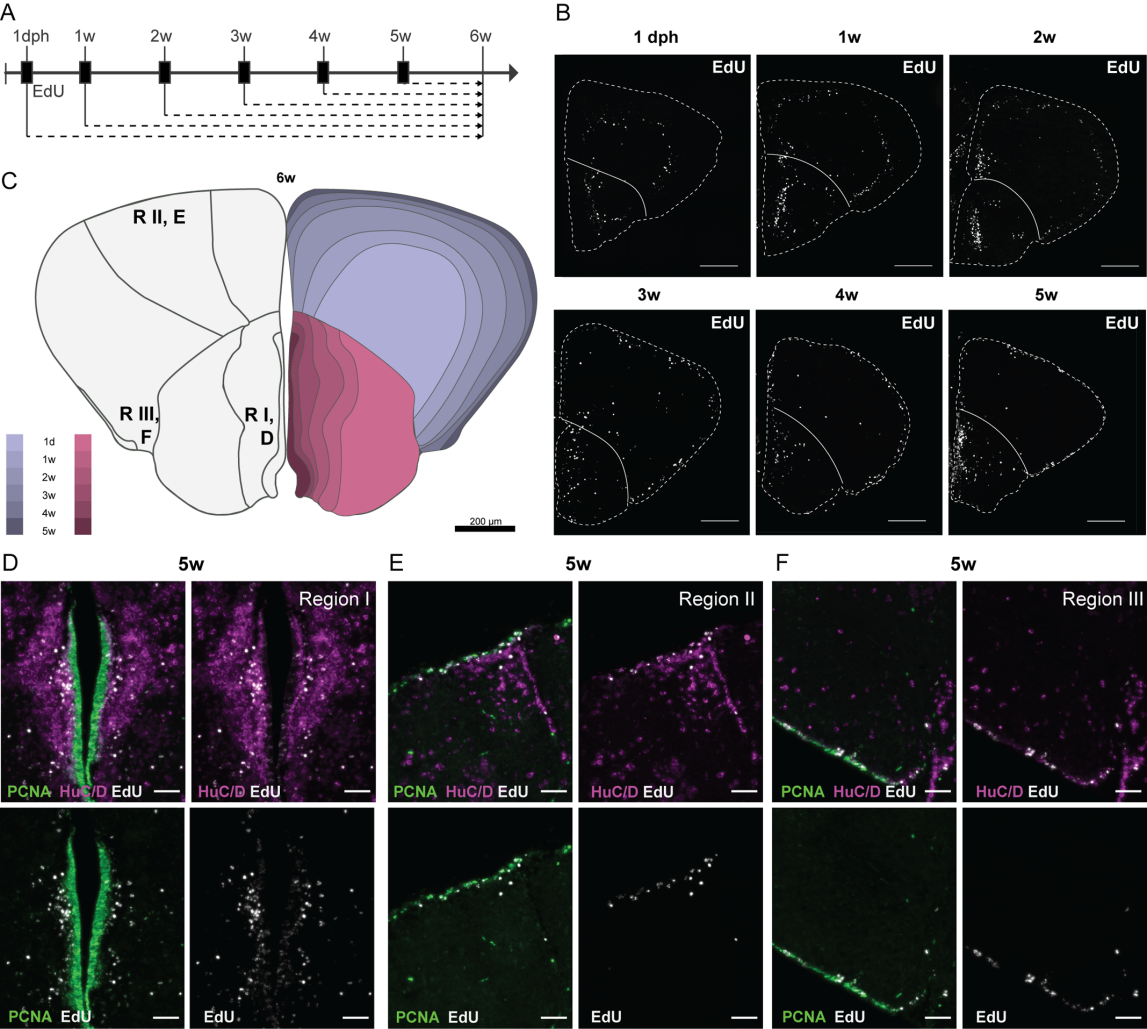
**EdU tracing reveals a neuronal stacking process during telencephalic growth.** (A) Design of the EdU birth-dating experiment. Each experimental group is exposed to 4 mM EdU for 16 h (black boxes) at different time points during development. EdU is incorporated into the DNA of dividing cells; their progeny are always traced until 6 weeks. (B) Distribution of EdU^+^ nuclei (white) on coronal sections at the mid-anterior-posterior level at 6 weeks. The timepoint of EdU administration is indicated above each panel. The outer border of the sections is indicated with a dashed line and the pallial-subpallial border with a solid line. In the pallium, EdU^+^ cells appear in concentric circles at differing distances from and parallel to the pallial surface. In the subpallium, the EdU^+^ cells appear more scattered, but the bulk of EdU^+^ cells seem to have migrated a comparable distance from the subpallial ventricular zone for each timepoint of EdU treatment. (C) Illustrative summary of the EdU tracing experiment. The opposite stacking process of the pallium and subpallium is color-coded for each period. The location of the pictures in D-F [neurogenic region (R) I, II, III] is indicated on the left hemisphere. (D-F) Zoom-in on EdU^+^ cells at each neurogenic region upon EdU treatment at 5 weeks. The EdU staining (white) is combined with an immunohistochemical staining for HuC/D (magenta) and PCNA (green). After 1 week of tracing from 5 weeks, the EdU signal is visible in the PCNA^+^ progenitor cells and HuC/D^+^ neurons at a distance from the ventricular zone. Note that region I contains more EdU^+^/HUC/D^+^ cells than regions II and III. A varying degree of EdU intensity is observed in the PCNA^+^ progenitors. Scale bars: 200 µm (B); 50 µm (D-F).

To understand the temporal growth pattern in the dorsal and ventral telencephalon, we performed a 5-ethynyl-2′-deoxyuridine (EdU) birth-dating experiment (Fig. 2A). The progeny of the dividing progenitors at the time of EdU pulse (1 dph, 1 weeks, 2 weeks, 3 weeks, 4 weeks and 5 weeks) was always visualized at 6 weeks of age (Fig. 2B). Cells born early after hatching (from 1 dph) were found in the parenchyme in a concentric circle parallel to the pallial surface, leaving the center of the 6 weeks pallial parenchyme devoid of EdU^+^ cells. Labeling at later ages revealed cells in consecutive circles at closer distances to the ventricular surface (Fig. 2B). This suggests that the center region of the pallium was already formed during embryonic development, similar to the dorsal pallium in zebrafish (Furlan et al., 2017). Subpallial growth occurred in a comparable consecutive manner. A dense line of EdU^+^ cells parallel to the subpallial neurogenic region could be identified, with the distance to the midline inversely related to the time of EdU tracing (Fig. 2B).

Systematic analysis of multiple coronal sections at the mid-anterior-posterior axis for each experimental group is summarized on top of the 6 weeks old telencephalic representation (Fig. 2C). Each layer demonstrates the estimated growth during this period for the pallium and subpallium separately. Neurogenesis seems to be additive from hatching and persists similarly into adulthood. We quantified the added surface areas at each traced stage as a percentage of the total surface at 6 weeks, using the EdU traced cells as a proxy for growth (Fig. S4A,B). In the pallium, the total area added (total growth) between 1 dph and 6 weeks was nearly half of the pallial surface. Most of that growth occurred between 1 dpf and 2 weeks. Added area decreased significantly over time in the pallium. In the subpallium, however, the added surface stayed relatively constant in the subpallium, again suggesting that subpallial growth was only marginal in this same time frame, relative to the pallium.

**Fig. 3.**
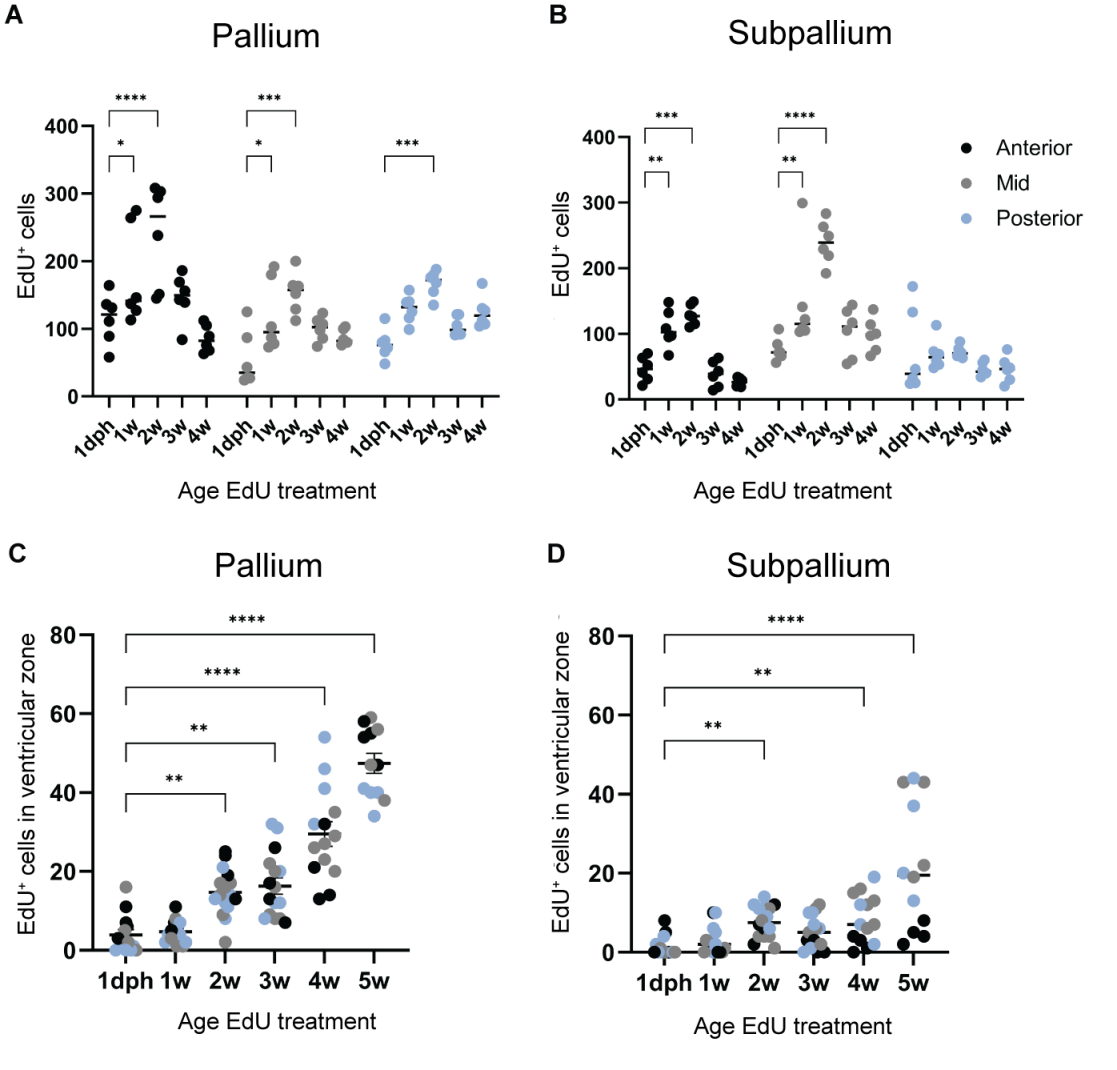
**Different pallial and subpallial growth dynamics identified by EdU tracing.** (A,B) The absolute number of EdU^+^ cells was quantified in 6-week-old coronal sections for both pallial (A) and subpallial (B) regions. Sections spanning the anterior-posterior axis were categorized into three levels: anterior, mid and posterior. Quantification was performed at each level for six individual hemispheres (*n*=6) from three fish per time point. Statistical analysis was conducted using a two-way ANOVA followed by post-hoc multiple comparisons against 1 dph. (C,D) To evaluate EdU label retention in progenitor cells, EdU^+^ cells located one to two cell bodies from the pallial ventricular surface (C) and subpallial midline (D) were quantified for each timepoint after tracing until 6 weeks. Quantifications were performed using individual hemispheres spanning the anterior-posterior axis. A minimum of two fish were used per time point (1 dph, *n*=14; 1 weeks, *n*=14; 2 weeks, *n*=16; 3 weeks, *n*=16; 4 weeks, *n*=14; 5 weeks, *n*=12). Statistical analysis was conducted using a Kruskal–Wallis test with post-hoc multiple comparisons against 1 dph. *P<0.05, **P<0.01, ***P<0.001, ****P<0.0001.

Still, the subpallial neurogenic region I is known to have the highest level of proliferation compared to pallial regions II and III (Tozzini et al., 2012; Ayana et al., 2024). EdU labeling at 5 weeks yielded strongly labeled cells at a distance from the progenitor zone in the subpallial region I, while in region II these cells were still found in the progenitor zone (Fig. 2D-F). Many PCNA^+^ progenitor cells seemed EdU^+^ in the subpallium. In the pallium, a lower number of HuC/D^+^/EdU^+^ neurons was present (Fig. 2E,F). To obtain a quantitative image of telencephalic growth, we quantified the number of EdU^+^ cells for each labeling time point at the anterior-mid and posterior level for the pallium and subpallium separately (Fig. 3A,B). The highest numbers of EdU^+^ cells were found for labeling at 2 weeks in both pallium and subpallium (peak of neurogenesis). Most EdU-traced cells were found in the anterior pallium (Fig. 3A,B, black dots) whereas most new cells accumulated at the mid-level in the subpallium (Fig. 3A,B, gray dots). We also quantified EdU^+^ cells in the ventricular zone (first two cell layers from the ventricle) in pallium and subpallium separately for the different labeling ages (Fig. 3C,D). In the pallium, numbers of EdU^+^ ventricular cells increased significantly from 2 weeks onwards, suggesting that newly formed cells do not leave the ventricular zone quickly. The situation in the subpallium is different, where a similar increase was only clear at 5 weeks (Fig. 3C,D). For the subpallium, our combined findings on marginal growth, maximal proliferation and less stalling of EdU^+^ cells at the ventricular surface are in line with the expectation that neurons born in the subpallial niche migrate to populate other brain regions, such as the olfactory bulbs and the pallium.

### Differential organization of RG cell subtypes in the developing telencephalon

**Fig. 4.**
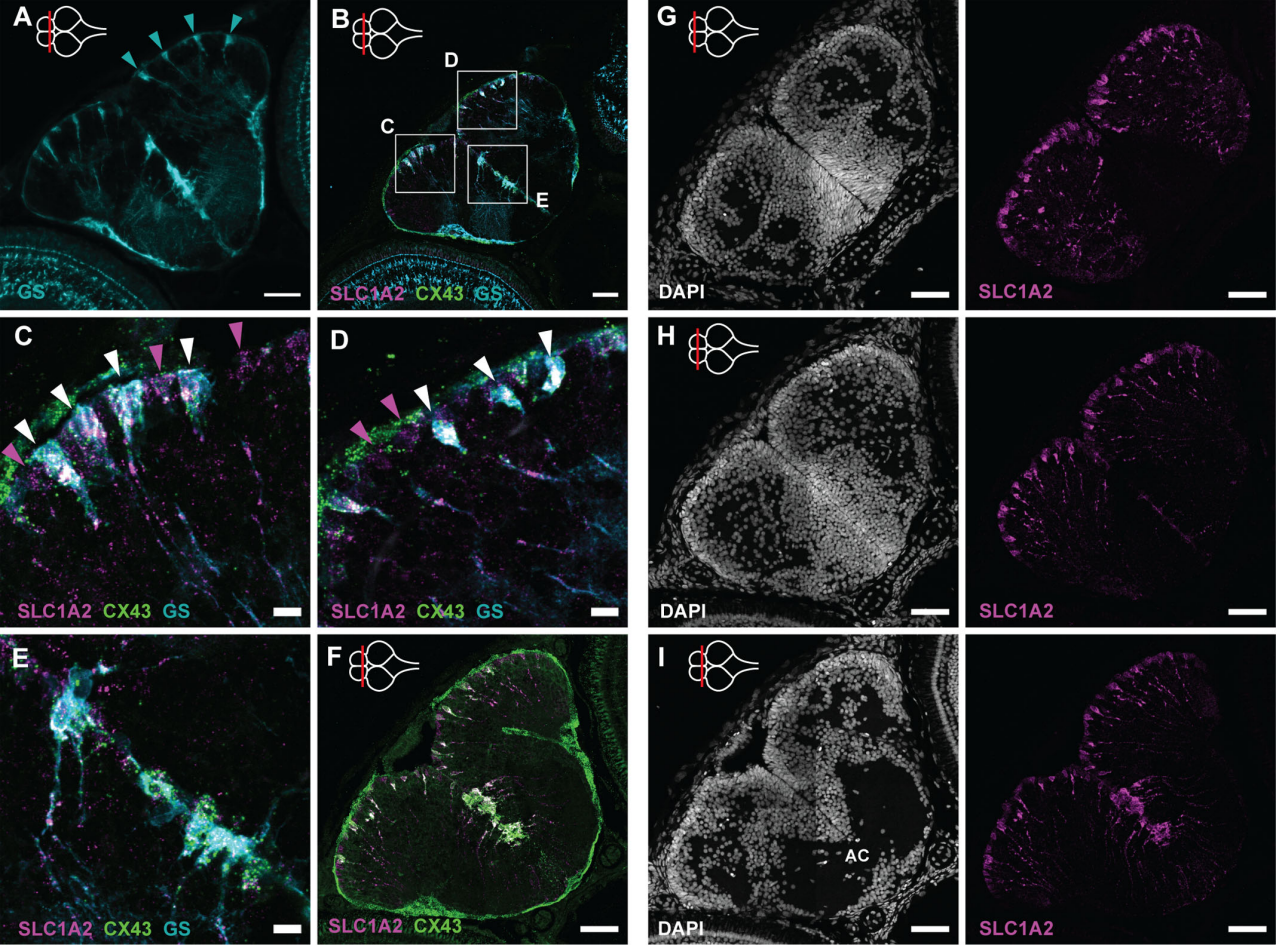
**Radial glia patterns in the developing telencephalon.** (A) Immunohistochemical staining of GS (turquoise) on a 5 dph telencephalon section at a posterior level. Isolated GS^+^ RGs are positioned one by one at the pallial surface (turquoise arrowheads) and in a dense cluster at the subpallial midline. (B) HCR-FISH targeting SLC1A2 (magenta) and CX43 (green) in combination with the immunohistochemical GS (turquoise) staining on a coronal section at the mid-anterior-posterior level. (C-E) Magnification of the squares in B. GS^+^/SLC1A2^+^/CX43^+^ RGs (white arrowheads) are interspersed with SLC1A2^+^-only RGs (magenta arrowheads), which are most probably not fully mature yet, at the pallial surface. (F) HCR-FISH targeting SLC1A2 (magenta) and CX43 (green) on a coronal section of the posterior telencephalon at 5 dph. At the midline, a dense cluster of CX43^+^SLC1A2^+^ RGs could be identified as the astroglia subcluster Astro-RG2. (G-I) Coronal sections of the telencephalon at 5 dph along the anterior-posterior axis. Left panels show the nuclear stain DAPI (white) to delineate the nuclear composition and density of the telencephalic domains. Right panels display HCR-FISH targeting SLC1A2 (magenta). Cell bodies, at the ventricular surface, and fiber structures, running towards the pia, are visible on all levels. The anterior-posterior position of the sections is indicated with a red line on a top-view illustration of the brain in the upper-left corner of the panels. Scale bars: 50 µm (A,B,F-I); 10 µm (C-E). AC, anterior commissure; HCR-FISH, hybridization chain reaction–fluorescent *in situ* hybridization.

Previous killifish studies used glutamine synthetase (GS) as a pan RG marker in the telencephalon (Coolen et al., 2020; Van houcke et al., 2021). To be able to delineate the RG subtypes as identified in the 6-week-old telencephalon by Ayana et al. (2024), we complemented our GS expression study with the analysis of the Astro-RG1/RG2 markers SLC1A2 (GLT1/EAAT2) and CX43 (Fig. S1A), and by relying on the knowledge that RG1 resides at the pallial surface and RG2 in subpallial niche I in a telencephalon at 6 weeks. At 5 dph, isolated GS^+^ cell bodies appeared tiled at the pallial surface with radial fibers extending to the pial surface of the dorsal pallium (Fig. 4A). Triple labeling of GS with SLC1A2 and CX43 showed that all GS^+^ cells are RG1 cells (Fig. 4B-D). In between such triple-positive RGs (Fig. 4C,D, white arrowheads), we could observe single SLC1A2^+^ cell bodies (magenta arrowheads). These may represent an immature or intermediate Astro-RG cell state. At 2 weeks, such GS^+^/SLC1A2^+^/CX43^+^ cell bodies appeared in a more dense group at the pallial surface. Yet, some immature single SLC1A2^+^ positive cells were still present in between these clusters of mature Astro-RG1s at the pallial surface (Fig. S5B,C). At the subpallial midline, we found a more dense cluster of GS^+^/SLC1A2^+^/CX43^+^ RGs already from 5 dph (Fig. 4E,F). In summary, a tiled RG-fiber pattern is visible from hatching in the telencephalon. At the subpallial midline, a dense cell cluster expressing Astro-RG2 markers is present from early development, while a mixture of mature and immature Astro-RG1s line the pallial surface.

An RG scaffold is essential for the proper neuroanatomical development of the telencephalon as RG fiber structures provide a scaffold for axon growth and neural migration to their final location in the whole network (Rakic, 1971; Kaur et al., 2020). At 5 dph and 2 weeks, a dense network of RG cell bodies and radial fibers was visible upon SLC1A2 mRNA detection along the anterior-posterior axis (Fig. 4G-I; Fig. S5A). The fibers of the subpallial Astro-RG2 followed a specific curvature into the lateral and ventral subpallium (Fig. 4F,I) and strongly resembled a subtype of adult zebrafish RG progenitors, with a more oval cell body and thick unbranched processes described before (März et al., 2010).

### Heterogeneity in the proliferative progenitor populations upon maturation

**Fig. 5.**
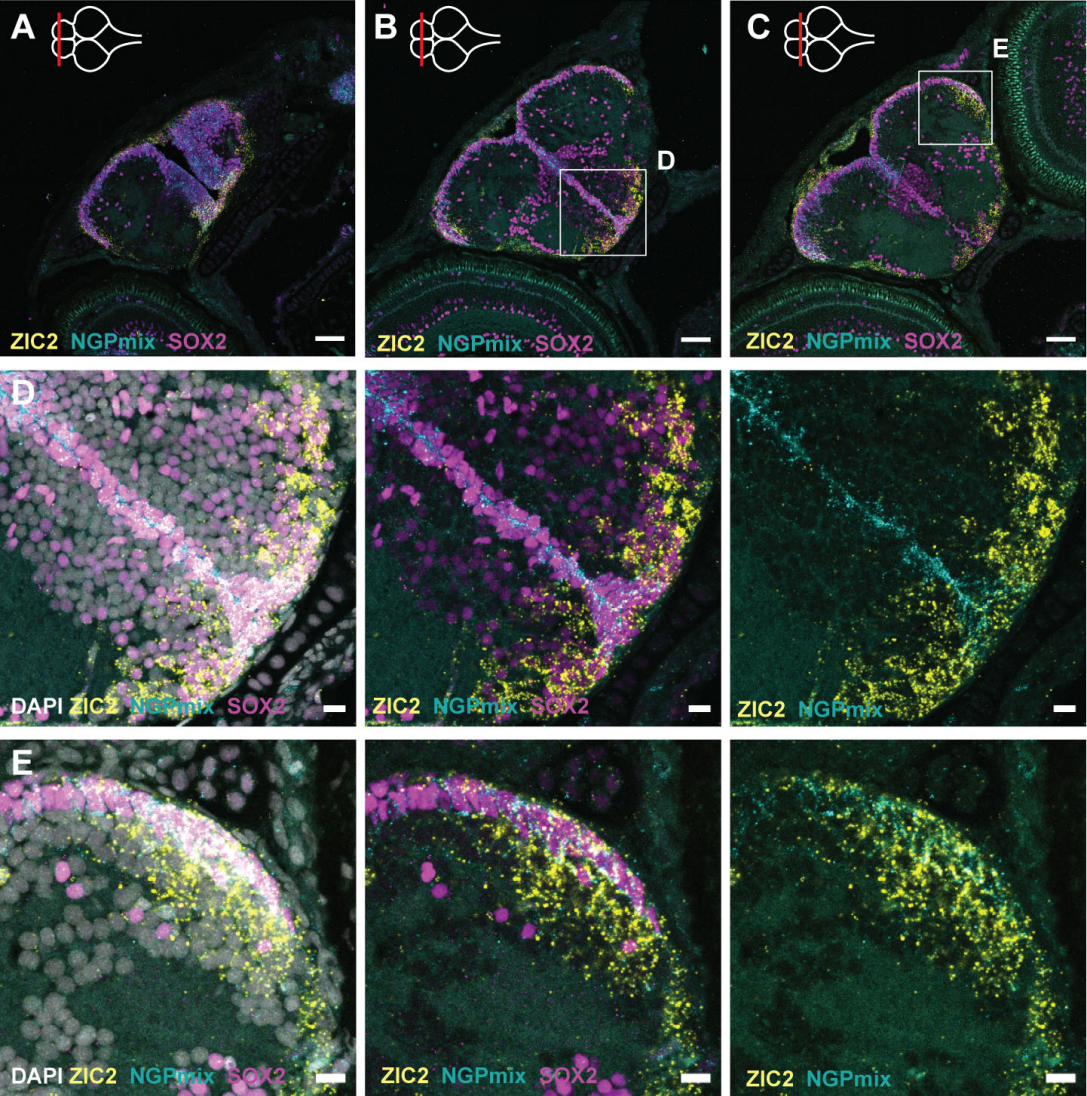
**Distribution of the non-glial and neuroepithelial-like progenitors.** (A-C) HCR-FISH targeting NGPmix (STMN1A+HMGB2A, NGPs, turquoise) and ZIC2 (NE-RGs, yellow) in combination with immunohistochemical staining for the progenitor marker SOX2 (magenta) on coronal sections along the anterior-posterior axis in the 5 dph telencephalon. (D,E) Magnifications of the regions in the boxes in B and C. The HCR-FISH targeting ZIC2 (yellow) and NGPmix (turquoise) and SOX2 staining (magenta) are combined with the nuclear stain DAPI (white). NGPs and NE-RGs are present in the subpallial midline and posterior pallial neurogenic regions. In the subpallium, both cell types retain their progenitor profile (SOX2^+^), while in the pallium, NE-RG3 loses its progenitor profile (SOX2^−^) in the parenchyme. Scale bars: 50 µm (A-C); 10 µm (D,E).

Next to the Astro-RG subpopulations, a ZIC2^+^ neuroepithelial RG subtype (NE-RG) was identified as the putative root stem cell cluster in the young-adult killifish telencephalon (Ayana et al., 2024). NE-RGs are located in subpallial neurogenic region I and pallial neurogenic region III (Ayana et al., 2024). Here, we confirmed that ZIC2^+^ NE-RGs were spatially restricted to these neurogenic regions at the ventral subpallial midline and at the posterior part of the lateral pallium from the start of post-embryonic development (Fig. 5A-C; Fig. S5D-G). In both regions, the NE-RGs expressed typical progenitor markers at the ventricular border (ZIC2^+^, SOX2^+^). Further into the parenchyme, these cells seemed to lose SOX2 expression in the pallium (ZIC2^+^, SOX2^−^) but not in the subpallium. (Fig. 5D,E). At the pallial-subpallial border, a dense cluster of SOX2^+^ cells was present in the parenchyme (Fig. 5B,C).

In the killifish telencephalon, the NGPs, and not the RG populations, are responsible for the bulk of proliferation from post-embryonic development until adulthood (Coolen et al., 2020; Van houcke et al., 2021; Ayana et al., 2024). In the developing post-embryonic killifish, we could identify the NGPs close to NE-RGs up until 2 weeks (Fig. 5; Fig. S5D-G). To ultimately prove a lineage relationship between these progenitor types, as suggested by this spatial proximity and the trajectory analysis in the young-adult killifish telencephalon (Ayana et al., 2024), a lineage tracing experiment would need to be performed.

**Fig. 6.**
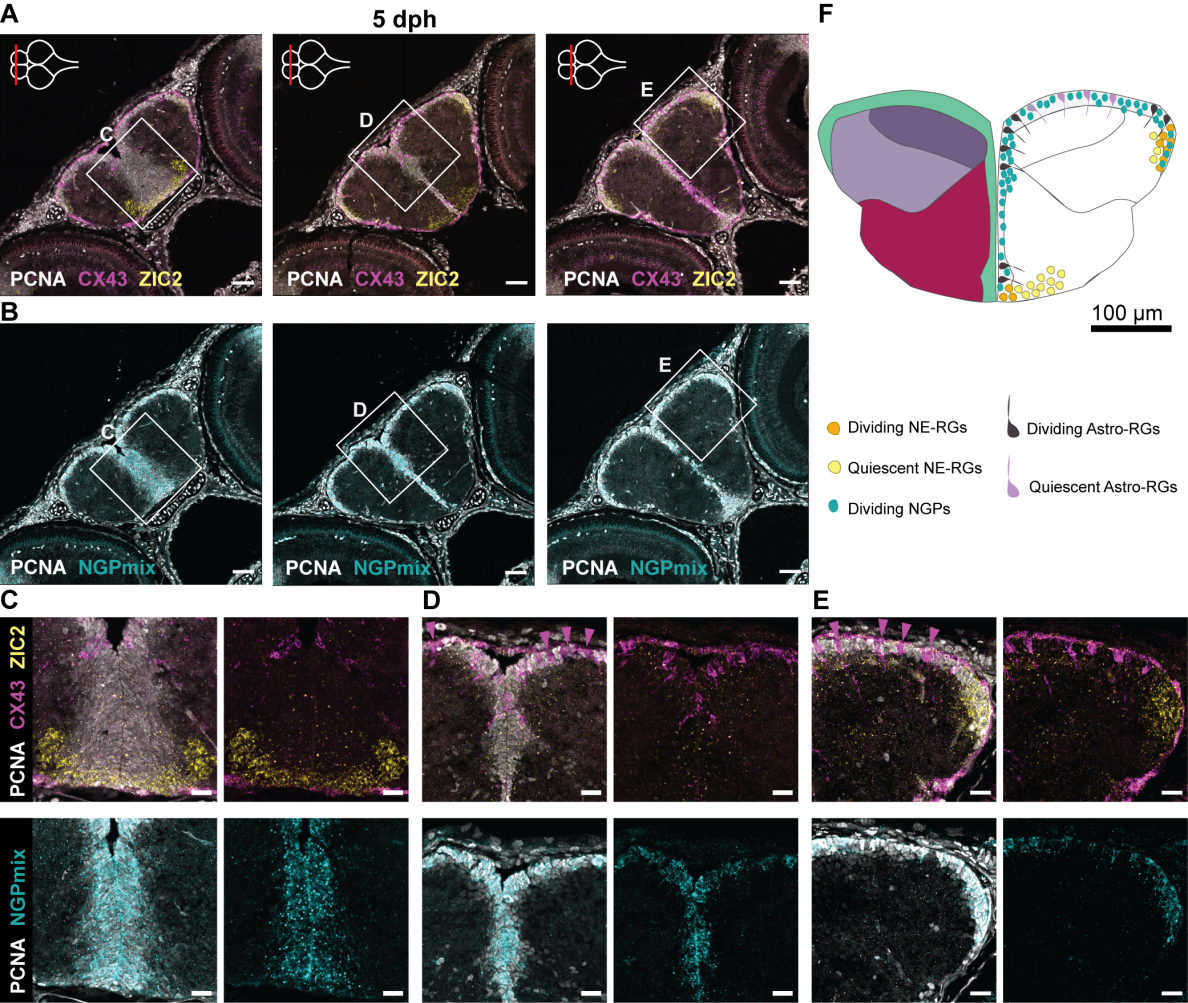
**Progenitor heterogeneity in the developing telencephalon.** (A,B) Adjacent coronal sections of the telencephalon at 5 dph along the anterior-posterior axis. The level of each section is indicated with a red line on the top-view brain illustration in the upper-left corner of each panel in A. HCR-FISH targeting CX43 (pan Astro-RG, magenta) and ZIC2 (NE-RG, yellow) (A) or NGPmix (turquoise) (B), is combined with immunohistochemical staining for the proliferation marker PCNA (white). NGPs account for the bulk of proliferation; Astro-RGs and NE-RGs appear both dividing and non-dividing depending on the location in the telencephalon. (C-E) Magnifications of the regions in the boxes in A and B. Non-dividing Astro-RGs (CX43^+^, PCNA^−^) are indicated with a magenta arrowhead and appear isolated in between stretches of dividing NGPs. (F) Illustration of a 5 dph coronal telencephalic section at mid-anterior-posterior level. The distribution of dividing and non-dividing Astro-RGs, NE-RGs and NGPs is displayed in the ventricular zone. Scale bars: 50 µm (A,B); 20 µm (C-E).

To characterize the proliferative profile of all major progenitor subtypes in the developing killifish telencephalon, we examined the spatial distribution of the proliferation marker PCNA (Fig. 6; Fig. S6). Previously, Coolen et al. (2020) investigated the proliferation capacity of GS^+^ RGs and SOX2^+^ GS^−^ NGPs in the developing killifish pallium. They identified a remarkable reduction of dividing GS^+^ RGs in the first 2 weeks of post-embryonic development. We probed for double labeling of PCNA with the Astro-RG-, NE-RG- and NGP-discriminating markers CX43, ZIC2, and the combination of STMN1A and HMGB2A (referred to as NGPmix), respectively, in the 1 dph, 5 dph and 2 weeks telencephalic pallium and subpallium (Fig. 6A,B; Fig. S6A,C). At 5 dph, we could observe a general co-labeling between NGPmix and PCNA, confirming the highly proliferative nature of NGPs in the developing telencephalon. In line, the NGPs were proliferative from 1 dph until 2 weeks in all three neurogenic regions (Fig. 6C-E; Fig. S6A-C). The NE-RGs at the pallial ventricular border were proliferative (ZIC2^+^, PCNA^+^), but the NE-RGs extending into the parenchyme did not express PCNA, in line with the absence of SOX2 expression in these cells (Fig. 5E and Fig. 6E; Fig. S6D). In the subpallium, we observed both PCNA^+^ and PCNA^−^ NE-RGs. Of note, the NE-RGs in the subpallium lost their proliferative signature (PCNA^−^) but kept SOX2 expression outside of the proliferative region (Fig. 5D and Fig. 6C). Comparable patterns of dividing and non-dividing NE-RGs were found during the first 2 weeks of development and are also comparable to previous findings in the young-adult telencephalon (Ayana et al., 2024).

The role of Astro-RGs in early brain growth (5 dph) seemed to be confined to one type of proliferating Astro-RGs (PCNA^+^, CX43^+^) that were mainly located at the dorsal midline and close to the posterior pallium, while non-proliferating Astro-RGs (PCNA^−^, CX43^+^) were located at the dorsal pallial surface (Fig. 6D,E). The posterior subpallial Astro-RG cluster, previously identified as Astro-RG2, also showed no proliferation (Fig. 6A). At 2 weeks, much fewer proliferating Astro-RGs were observed, also at the dorsal pallial midline (Fig. S6C,E). In summary, our dataset confirms that, in early life, the killifish progenitor pool exists out of the highly proliferative NGPs, and identified novel populations of dividing and quiescent NE-RGs in specific niches, while distinguishing subpallial and pallial Astro-RG progenitor populations that decline their proliferation shortly after hatching (Fig. 6F).

### Intermediate progenitors in the pallial ventricular region

**Fig. 7.**
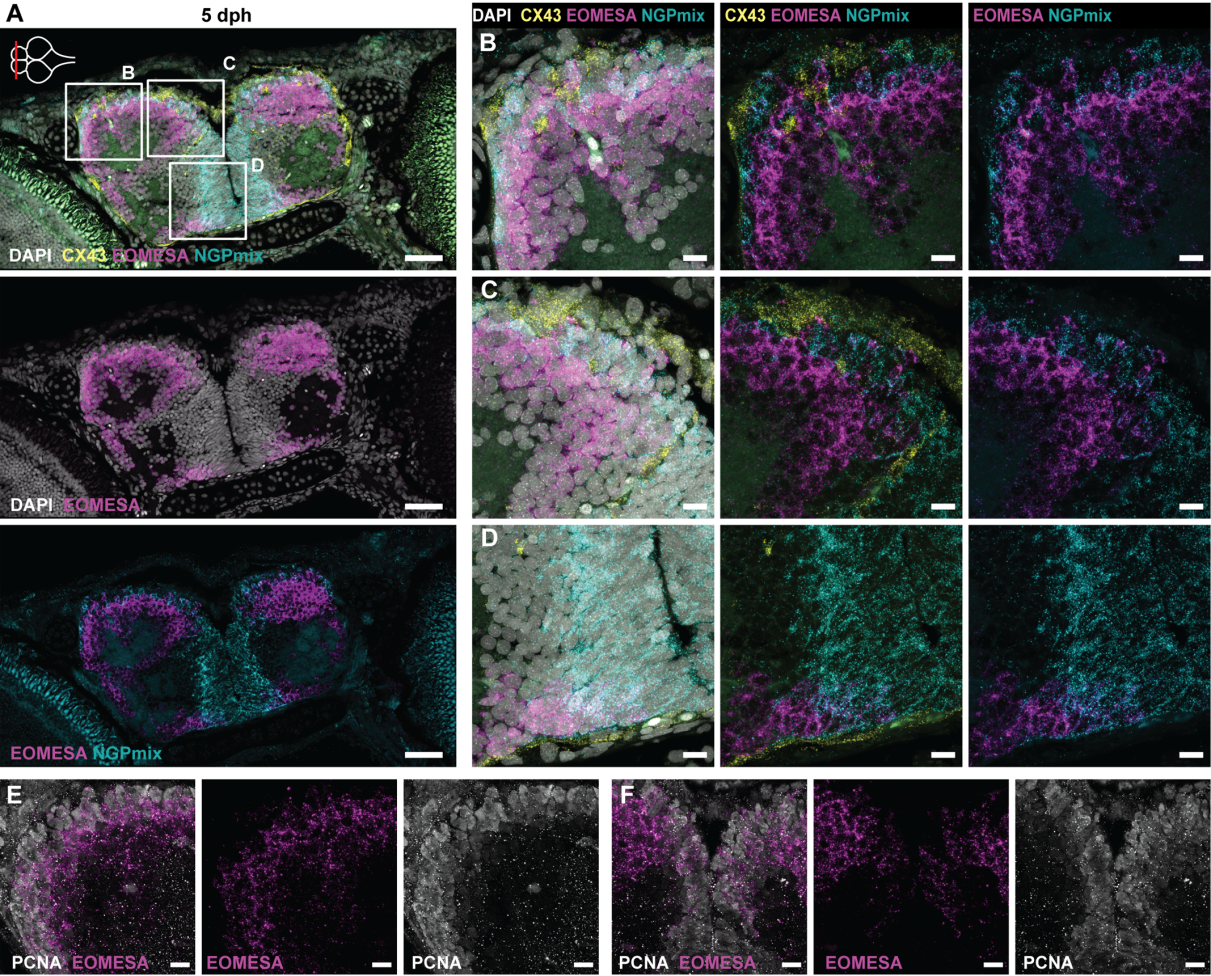
**Intermediate progenitors in the killifish pallium.** (A) HCR-FISH targeting CX43 (Astro-RGs, yellow), NGPmix (turquoise) and the immature excitatory neuron marker EOMESA (magenta) in combination with the nuclear stain DAPI (white) on a coronal section of the telencephalon at 5 dph. The anterior-posterior level is depicted with a red line on the top-view brain illustration in the upper-left corner. (B-D) Magnifications of the regions in the boxes in A. Intermediate progenitors (EOMESA^+^/NGPmix^+^) are found at the pallial neurogenic region and ventral subpallium, dispersed in between Astro-RGs (only in pallium) and NGPs. (E,F) HCR-FISH targeting EOMESA (magenta) in combination with immunohistochemical staining for the proliferation marker PCNA (white). The panels are magnifications of zones on a section of a comparable anterior-posterior level as in A. E and F are magnifications of the lateral pallium and dorsal midline, respectively. Proliferating PCNA^+^/EOMESA^+^ progenitors are present at the midline and pallial surface. Scale bars: 50 µm (A); 10 µm (B-F).

Intermediate progenitors are RG daughter cells in the embryonic mouse cerebral cortex and are an important secondary proliferating progenitor population generating the bulk of excitatory glutamatergic projection neurons. These intermediate progenitors specifically express EOMESA (TBR2) (Hevner, 2019). In the adult zebrafish telencephalon, expression of EOMESA is observed in most parenchymal neuronal zones and at the pallial ventricular layer (Ganz et al., 2015). Here, we explored the presence of EOMESA^+^ progenitors in the neurogenic niches of the developing killifish telencephalon. EOMESA^+^ cells could be found at the ventricular border of the pallial surface (Fig. 7A-D). To investigate whether these cells were direct descendants of NGP or RG cells, we reasoned that we would find marker co-expression in a portion of these cells if they shared the lineage. Double labeling with the NGP markers (STMN1A and HMGB2A) revealed co-expression of EOMESA and NGPmix in the neurogenic niches (Fig. 7B-D). This was particularly visible at the pallial midline in the telencephalon at 2 weeks, where EOMESA expression did not extend into the parenchyme but was restricted to these cells at the ventricular border (Fig. S6A-C). We additionally probed for co-expression of the Astro-RG marker CX43 and EOMESA but could not detect any. Still, EOMESA^+^ cells were found in close proximity to CX43^+^ Astro-RGs (Fig. 7B-D; Fig. S7B,C). So, EOMESA^+^/NGPmix^+^ intermediate progenitors could be found in the pallial ventricular regions, intermingled with NGPs (NGPmix^+^) and Astro-RGs (CX43^+^). In the anterior subpallium, we could also observe EOMESA^+^ cells at the outer border of the ventral NGP-rich neurogenic region (Fig. 7D; Fig. S7A). Since intermediate progenitors have the capacity to divide a limited number of times before differentiating into neurons, we verified the proliferation profile of these EOMESA^+^ cells in the neurogenic regions. Double labeling with the proliferation marker PCNA confirmed that the EOMESA^+^ cells in the progenitor regions of the telencephalon can proliferate at hatchling and juvenile stages (Fig. 7E,F; Fig. S7D,E). Our results suggest that the EOMESA^+^ cells in the developing killifish telencephalon might be intermediate progenitors that arise from NGPs; however, lineage tracing experiments are needed to prove such a developmental link.

### Development and organization of inhibitory and excitatory neurons

To visualize where neuronal subtypes are born in the post-embryonic killifish, we probed for the presence of two main neuronal classes: gamma-aminobutyric acid-releasing (GABAergic) neurons and glutamatergic neurons, using the canonical markers GAD1B and SLC17A7 (VGLUT1), respectively. To examine the maturation state of these post-mitotic cells, we combined this with analysis of the immature inhibitory and excitatory neuron markers DLX1 and EOMESA, respectively. DLX1 is known to be an important transcription factor for neuronal differentiation and migration of GABAergic neurons (Cobos et al., 2007).

**Fig. 8.**
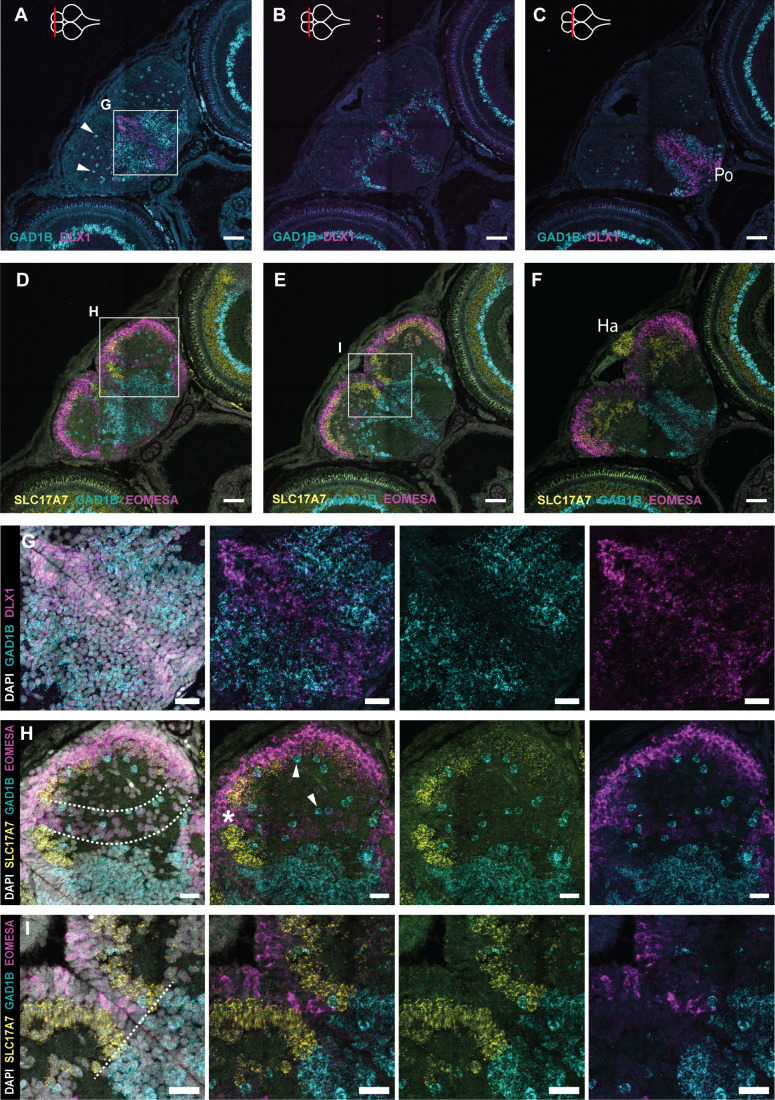
**Excitatory and inhibitory neurons in the 5** **dph telencephalon.** (A-C) Coronal sections of the telencephalon at 5 dph along the anterior-posterior axis. HCR-FISH targeting DLX1 (magenta) and GAD1B (turquoise) mRNA expression shows the distribution of immature and mature inhibitory neurons, respectively. (D-F) Adjacent coronal sections of A-C; HCR-FISH targeting EOMESA (magenta), SLC17A7 (yellow) and GAD1B (turquoise) mRNA expression shows the distribution of immature and mature excitatory neurons, and the mature inhibitory neurons, respectively. The anterior-posterior position of the sections is indicated with a red line on the top-view brain illustration in the upper-left corner of A-C. The border of the telencephalon (posterior-most section, C,F) is recognizable based on the presence of the habenula on top of the dorsal pallial surface, positive for SLC17A7. (G) Magnification of the region in the box in A. HCR-FISH labeling DLX1 and GAD1B in combination with a nuclear stain (DAPI, white) to visualize all cell bodies. Progenitor cells at the midline are negative for DLX1 (DAPI^+^). (H,I) Magnifications of the regions in the boxes in D and E. HCR-FISH labeling EOMESA, SLC17A7 and GAD1B in combination with DAPI. The progenitor cells at the ventricular surface are negative for EOMESA (DAPI^+^). (H) A stream of low-level EOMESA^+^ cells is visible (area between the dotted lines), stretching from medial to ventrolateral pallium. This band is devoid of SLC17A7^+^ expression (asterisk). (I) A clear border is discernable between inhibitory (GAD1B^+^) and excitatory (SLC17A7^+^ and/or EOMESA^+^) cells around the midline, indicated with a dotted line. GAD1B^+^ cells are found scattered throughout the pallium (white arrowheads). (A-F) Scale bars: 50 µm (A-F); 20 µm (G-I). Ha, habenula; Po, preoptic area.

DLX1^+^ cells arise next to the NGP-rich neurogenic region in the subpallium (Fig. 8A-C). DLX1^+^/GAD1B^+^ and DLX1^−^/GAD1B^+^ inhibitory neurons were heavily populating the ventral portion of the rostral telencephalon (Fig. 8A,G). GAD1B^+^ cells could also be found scattered throughout the pallium. The absence of DLX1^+^ cells in the pallial neurogenic niches indicated that these GAD1B^+^ GABAergic neurons were likely formed in the subpallium and might have arrived in the pallium via tangential migration. In the mid-posterior telencephalon (Fig. 8B), a comparable pattern of DLX1^+^ and GAD1B^+^ neurons could be observed, albeit at a lower density. Of note, the posterior subpallium showed a lower density of cell bodies in general (Fig. 1B). GAD1B^+^ cells were also found at the lateral border of the mid-posterior subpallium. At the posterior edge of the telencephalon, the ventral subpallium (preoptic area) showed a high density of DLX1^+^ and GAD1B^+^ cells at the midline (Fig. 8C).

EOMESA^+^ neurons could be observed at the periventricular zone of the pallium along the anterior-posterior axis (Fig. 8D-F; Fig. S8A-C). More mature excitatory neurons (SLC17A7^+^) were positioned at the parenchymal side of the immature EOMESA^+^ neurons, in line with the stacking growth model of the pallium. The neural progenitor cells at the midline and ventricular surface, lacking neuronal markers, can be clearly distinguished from the cells committed to the neuronal lineage (Fig. 8G,H). Anteriorly, we could observe a stream of EOMESA^+^ neurons extending from the medial pallium to the posterior pallial region (Fig. 8H, in between the dotted lines). This stream was devoid of mature excitatory neurons (Fig. 8H, asterisk), but GAD1B^+^ neurons were scattered throughout. The pallial-subpallial border could be distinguished by the clear expression boundary between inhibitory and excitatory cells at the midline (Fig. 8I). Already at 1 dph, GAD1B^+^ mature inhibitory neurons could be found near SLC17A7^+^ mature excitatory neurons (Fig. S8D). At this early-life stage, larger areas with EOMESA^+^ and SLC17A7^+^ overlap indicated differentiating excitatory neurons in the pallium (Fig. S8A-D). In sum, the early post-hatching killifish has a typical teleost telencephalon with glutamatergic neurons generated at the pallial surface and GABAergic cells formed in the DLX1^+^ subpallium ventricular zone.

## DISCUSSION

The African turquoise killifish is an extensively used model for aging research in a broad spectrum of research fields, including genetics, evolution, immunology and neurobiology. We and others have exploited its conveniently short lifespan to study the aging process of the central nervous system, emphasizing the impact on neuro(re)genesis. Here, we probed when the progenitor population sustaining adult neurogenesis in the killifish telencephalon arises, and how the explosive growth process during early post-hatching development takes place. We charted the sequential generation of neural cells and telencephalon morphogenesis from hatchling to young-adult stages and identified (1) a distinct stacking process of newborn cells in the pallium and subpallium, (2) a more pronounced lateral growth of the pallium compared to the subpallium, (3) the presence of a maturing scaffold of RG nature early post-hatching, (4) a high proliferative activity during the first 2 weeks after hatching, and a region-dependent decrease in proliferation over time, and (5) a clear pallial-subpallial patterning of progenitor cells committed to either the glutamatergic or GABAergic neuronal lineage. The spatial map presented here shows the diversity of progenitor cells, constructed during development and maintained in adulthood, and will be indispensable in future studies on lineage relationships, and age-related decline in neurogenesis and regeneration.

Despite differences in the timing of embryonic and post-embryonic development, the morphogenesis of the telencephalon in killifish and zebrafish is at a comparable stage upon hatching. Birth-dating studies in zebrafish have shown that the center region of the pallium is already formed during embryonic development, which aligns with our findings for killifish (Furlan et al., 2017). A study on whole-body growth of GRZ killifish reported accelerated growth in the first 4 weeks after hatching, with a peak in the second week (Blažek et al., 2013). Quantification of pallial and subpallial growth, visualized with EdU birth dating, revealed that most cells were generated in the first 2 weeks. In both zebrafish and killifish, lateral expansion of the pallium only begins after hatching and becomes prominent around 2 weeks after hatching (Dirian et al., 2014). Because of the explosive growth of the killifish in early life, the pallial surface expands three times faster compared to that of zebrafish in the first 2 weeks after hatching (Coolen et al., 2020). Measurements of the dorsoventral and mediolateral growth of the telencephalon also revealed that, from 2 weeks to young-adult (6 weeks), the pallium still doubled in size on average in all directions measured. For the subpallium, this difference was smaller. This additional growth might be attributed to the increase in neuropil over this period.

The EdU tracing experiment revealed distinct neurogenic dynamics for the pallium and subpallium. Quantification discovered that peak growth occurs more anteriorly in the pallium than the subpallium, and that, after 2 weeks, fewer subpallial progenitors (located at the ventricular midline) retain EdU label. Comparable experiments in other teleost models mainly focused on the pallial growth. In zebrafish, a similar stacking process of the pallium from embryonic to juvenile stages was revealed, using a Tet-On birth-dating strategy to specifically trace the progeny of HER4^+^ RGs (Furlan et al., 2017). In teleost fishes that have an inverted telencephalon, the central pallial core, created during embryogenesis, thus becomes surrounded by concentric layers of newborn cells generated sequentially over time. In zebrafish, HER4^+^ RGs produce the majority of newborn neurons (Furlan et al., 2017). In the developing zebrafish, however, two neural progenitor lineages are identified in the pallium. One emerges from HER4^+^ RGs at the dorsomedial pallium, giving rise to neurons and new RGs. The other originates at the lateral pallium and has a neuroepithelial and amplifying character, which thus resembles the NE-RG progenitors discovered in killifish (Dirian et al., 2014; Ayana et al., 2024). Than-Trong et al. (2020) also described a HER4^−^ progenitor pool as the source of the gradual expansion of the RG population in the adult zebrafish. While HER4^+^ (HES5) RGs are also found in killifish telencephalon, expansive growth is supported by NGPs in all neurogenic regions during post-embryonic development and in the adult killifish telencephalon (Coolen et al., 2020; Ayana et al., 2024). The spatial proximity of NGP and NE-RG marker expression at the subpallial midline and posterior pallium is prominent around hatching (1-5 dph). Lineage tracing from NE-RG3 and NGPs will be necessary to identify the exact progeny of these cell types and renewal of the progenitor pool during development, adulthood and aging.

A scaffold-like structure of radial fibers is already present from hatching in the killifish telencephalon. The non-overlaying radial structure is reminiscent of tiling of RGs and astrocytes in the developing and mature mouse brain (Bushong et al., 2002; Nakagawa et al., 2019). Tiling of glial cells, well described in mammals for cortical astrocytes, is a conserved mechanism already present in invertebrates (Baldwin et al., 2021; Pogodalla et al., 2022). Coolen et al. (2020) identified a strong decrease in proliferating RGs at the dorsal surface of the killifish pallium. Our data revealed that these RGs represent the pallial Astro-RG type, Astro-RG1. We confirmed the decrease in proliferating Astro-RG1s and discovered an uneven distribution of dividing and non-dividing pallial Astro-RG1s. The dividing Astro-RG1s seem to be confined to the dorsal midline and posterior pallium, but their number is relatively limited and the bulk of proliferation comes from the NGPs.

Using deterministic markers (DLX1, EOMESA, GAD1B, SLC17A7), we could visualize the maturation sequence of excitatory and inhibitory neurons in the killifish telencephalon. At 1 dph, mature GABAergic neurons densely populated the subpallium and were scattered throughout the pallium, whereas in zebrafish, they are only observed in the posterior pallium at a similar age (Mueller et al., 2006). Neurons from the GABAergic lineage are exclusively generated in subpallial neurogenic Region I. Progenitors and immature neurons committed to the glutamatergic lineage were identified in both the pallial and subpallial neurogenic regions. Subpallial EOMESA^+^ cells were spatially restricted to the anterior-ventral-most part of the telencephalon. In other teleost species, the ventral and lateral nuclei of the anterior subpallium are thought to be homologous to the septum and known to have expression of EOMESA (Wullimann and Mueller, 2004; Mueller et al., 2008). Even though the timing of both embryonic and post-embryonic development is considerably different for zebrafish and killifish, we observed a comparable spatial organization of inhibitory and excitatory neurons for the telencephalon once hatched (Wullimann and Mueller, 2004; Mueller et al., 2006, 2008, 2011; Ganz et al., 2015). TBR2 (EOMESA) shows comparable expression patterns in the developing pallial regions of both species (Mueller et al., 2008). In the killifish pallium, glutamatergic neurons are produced along the entire dorsal surface. Our study reveals the neuronal diversity in the developing killifish telencephalon by spatially delineating the immature and mature excitatory and inhibitory neurons. In the young-adult killifish telencephalon, single-cell sequencing revealed a more extensive diversity of cell types, including 12 excitatory and five inhibitory neuronal clusters (Ayana et al., 2024). The advent of large-scale single-cell studies is likely to discover an even more impressive diversity, such as the recently generated neuronal cell type atlas of the goldfish telencephalon, generated by combining single-cell RNA sequencing and spatial transcriptomics, which uncovered 88 GABAergic and glutamatergic neuronal subclusters (Tibi et al., 2023). In conclusion, we have revealed the cellular diversity during explosive growth of the killifish telencephalon, uncovering specific progenitor signatures for each neurogenic region and evidence for distinct glutamatergic and GABAergic progenitor lineages in the post-embryonic telencephalon.

## MATERIALS AND METHODS

### Animals and housing

All experiments were performed with African turquoise killifish (*N. furzeri*) from the inbred GRZ-AD strain. When the embryos were ready to hatch (Golden Eye stage), the eggs were moved to a hatching tank with a small volume of ice-cold hatching solution (Humic acid, Sigma-Aldrich, 53680, 1 g/l in aquarium water) with a continuous flow of oxygen (Polačik et al., 2016; Van houcke et al., 2021). The fish were raised in the hatching tank for 1 week at 26°C with a daily addition of fresh aquarium water. After the first week, juvenile fish were transferred to a 3.5 l aquarium in a zebTEC Multi-Linking Housing System (Techniplast) and housed under standardized conditions: temperature 28°C, pH 7, conductivity 600 μs, 12 h/12 h light/dark cycle. After hatching, the fish were fed twice a day with *Artemia salina* (Ocean Nutrition). From 4 weeks onwards, they were fed with *Artemia salina* (Ocean Nutrition) and mosquito larvae (*Chironomidae*). All experiments were approved by the KU Leuven ethical committee (025/2021; 155/2023), under the European Communities Council Directive of 20 October 2010 (2010/63/EU).

### EdU labeling

To label dividing cells and trace the progeny, batches of fish (*n*=3-6) were placed in EdU (4 mM) for 16 h. Each group was pulsed once with EdU at a specific age: 1 dph, 1 week, 2 weeks, 3 weeks, 4 weeks and 5 weeks. All fish were sacrificed at the age of 6 weeks (Fig. 2A).

### Tissue collection and processing

Hatchlings (1-5 dph), juveniles (2 weeks) and young-adult fish (6 weeks) were euthanized in 0.1% buffered tricaine (MS-222, Sigma-Aldrich). Hatchling and juvenile tissue (full body) were fixed overnight in 4% paraformaldehyde [PFA; 8.18715, Sigma-Aldrich, in phosphate-buffered saline (PBS)] at 4°C. After euthanasia, young-adult fish were perfused with PBS and 4% PFA (Mariën et al., 2022). The brains were dissected and fixed overnight in 4% PFA at 4°C. Next, the tissue (full body or brain) was saturated overnight in 30% sucrose (in PBS) after which the tissue was embedded in 30% sucrose and 1.25% agarose in PBS. Coronal sections (10 µm thick) were cut with a CM3050s cryostat (Leica), and the sections were collected on SuperFrost Plus Adhesion slides (10149870, Thermo Fisher Scientific). The sections were stored at −20°C until hybridization chain reaction (HCR), immunohistochemistry (IHC) or EdU staining.

### HCR

The probe pools used for HCR were generated and validated as described in detail before (Deryckere et al., 2021; Van houcke et al., 2021; Elagoz et al., 2022; Styfhals et al., 2022) and ordered via Integrated DNA Technologies (IDT). The HCR protocol (HCR v3.0), based on the protocol of Choi et al., (2018), is adapted for cryosections as described in Van houcke et al. (2021), without the proteinase K permeabilization step. To optimize the signal-to-noise ratio, the probe pool concentration ranged between 0.3 and 1.8[Supplementary-material sup1] pmol, depending on the target (Table S1). In case the amplifier (Molecular Instruments) was linked to Alexa Fluor 546, the sections were mounted with SlowFade Gold Antifade Mountant (S36936, Invitrogen) to improve the photostability of the fluorophore. When combining HCR with IHC, the slides were washed three times with 5xSSCT (0.1% Tween 20 in saline-sodium citrate buffer) after the hybridization and amplification steps, before proceeding with the IHC protocol.

### IHC

The cryosections were first dried for 30 min at 37°C before proceeding with wash steps in TBS (0.1% Triton-X-100). The tissue was blocked with 20% normal donkey serum (D9663, Sigma-Aldrich) in Tris-NaCl blocking buffer (TNB). After blocking, the sections were incubated overnight with the primary antibody diluted in Pierce Immunostain Enhancer (46644, Thermo Fisher Scientific) (Table S2). After wash steps in TBS, the sections were incubated with the secondary antibody in TNB for 2 h. Afterwards, the sections were rinsed in PBS followed by nuclear staining with 4′,6-diamidino-2-fenylindool (DAPI; 1:1000 in PBS, 32670, Sigma-Aldrich). All sections were mounted with Mowiol and a glass cover slide. Whenever IHC was preceded by an HCR, all wash steps were performed with PBS-T (0.1% Tween 20 in PBS) instead of TBS.

### EdU staining

EdU staining was performed using the Click-iT™ EdU Cell Proliferation Kit for Imaging (C10340, Invitrogen). Briefly, the sections were dried for 30 min, rinsed in PBS and permeabilized in 0.5% Triton X-100 in PBS. After an additional wash step in 3% bovine serum albumin (BSA; in PBS), the Click-iT^®^ reaction cocktail was incubated for 30 min in the dark. Afterwards, sections were rinsed with 3% BSA, and a nuclear stain with DAPI was performed. If the EdU staining was combined with IHC, the EdU staining preceded the IHC. The sections were mounted with Mowiol and a glass cover slide.

### Imaging

Images were acquired with a widefield (Axio Observer7, ZEISS) or confocal (LSM 900 with Airyscan 2, ZEISS) microscope. The images were further processed with ZEN 3.7 software (ZEISS).

### Delineation of telencephalic levels and regions

To analyze and categorize the telencephalon into three distinct levels, anterior, mid and posterior sections were selected based on anatomical landmarks and the distance in micrometers between consecutive sections. This approach provided a robust and consistent means of identifying comparable anterior-posterior levels across developmental stages (1 dph, 5 dph, 2 weeks, 6 weeks). Using this approach, the complete anterior-posterior axis of the telencephalon was probed, as illustrated in Fig. S3B.

The approximate distances between selected sections in Fig. 1 and Fig. S1 are as follows (anterior to posterior): 1 dph, 50 µm; 5 dph, 50-100 µm; 2 weeks, 100 µm; 6 weeks, 120-180 µm. Anatomical landmarks were used to refine section selection and ensure alignment between stages. Anterior sections were identified by the presence of the olfactory bulb. Mid-level sections were characterized by the progenitor region spanning the dorsal to ventral surface and the presence of a ventrolateral neuronal cluster. Posterior sections were defined by the appearance of a central circular neuronal cluster in the pallium, comparable to the central zone of the dorsal pallium as described in the adult killifish telencephalon (D'Angelo, 2013). To minimize potential deviations caused by cutting angle differences, levels were determined independently for each hemisphere during quantifications. Small deviations in cutting angle were evident as apparent asymmetries, particularly in earlier developmental stages.

Pallial and subpallial regions were distinguished based on marker analysis of HuC/D and PCNA, complemented by patterns identified using excitatory and inhibitory neuron markers (Fig. 8). Distinct spatial changes in progenitor and neuronal cluster organization facilitated the precise delineation of pallial and subpallial regions along the anterior-posterior axis.

### Quantification and statistical analysis

Area fraction (%) measurements of SV2 staining (Fig. S2C) were quantified using ImageJ (Fiji, version 1.53f51) with a consistent threshold applied to all images prior to analysis. Telencephalon distances (Fig. S3A) were measured using the Line tool in ZEN 3.7. Quantification of EdU signal (Fig. 3) and growth measurements (Fig. S4) were conducted using the Contour and Events tools, respectively, in ZEN 3.7.

All statistical analyses were conducted using GraphPad Prism software (v9.0.0). Data are presented as mean±s.e.m. Statistical significance was defined as *P*≤0.05. Statistically significant differences are denoted by asterisks above the respective data points in the graphs. Depending on the data, a Kruskal–Wallis test or a two-way ANOVA was performed, followed by post-hoc multiple comparisons.

## Supplementary Material

10.1242/biolopen.061984_sup1 Supplementary information
