# Supplementary material for: Early-life growth and cellular heterogeneity in the short-lived African turquoise killifish telencephalon
Source: Biol Open. 2025 Apr 22;14(4):bio061984. doi: 10.1242/bio.061984 (PMC12045632; doi:10.1242/bio.061984)
Supplement: Supplementary information [file biolopen-14-061984-s1.pdf]

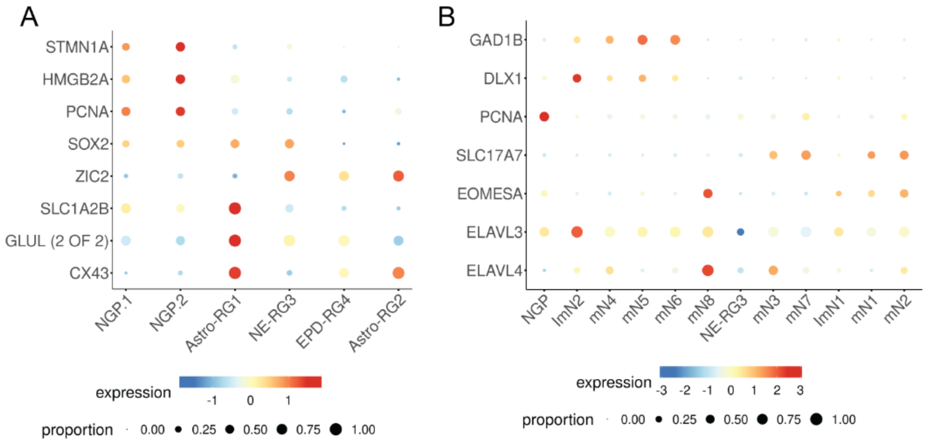

Fig. S1. Marker expression as determined in young-adult 6-week-old killifish telencephalon (Ayana, Zandecki et al., 2024). Dot plots show markers used to identify (A) radial glia subtypes and non-glial progenitors, and (B) immature (ImN) and mature (mN) inhibitory (from left to right: clusters 2-5) and excitatory (clusters 6, 8-12) neuron clusters. Dot size represents the percentage of cells expressing the gene (0–100%), while the color indicates the relative expression level. Data were obtained from the online killifish telencephalon single-cell RNA sequencing database published by Ayana, Zandecki et al. (2024).

Biology Open • Supplementary information

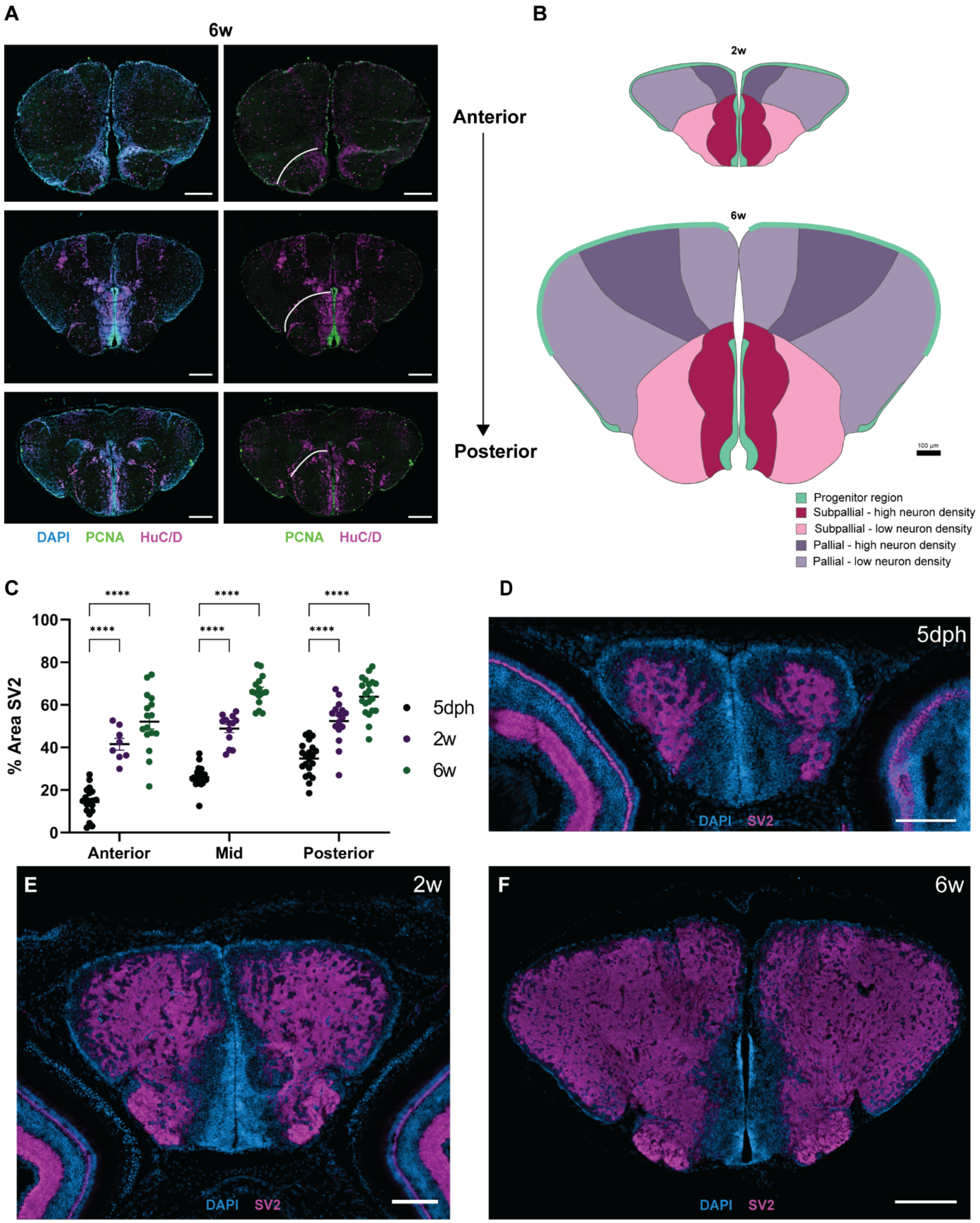

Fig. S2. Explosive growth of the young-adult telencephalon. (A) Immunohistochemical staining for PCNA (green) and HuC/D (magenta) in combination with a nuclear stain (DAPI, blue) on coronal brain sections. Three levels alongf the anterior-posterior axis are shown from a 6w old killifish, from anterior to posterior. The white line represents the pallial-subpallial border. **(B)**

Biology Open • Supplementary information

On-scale illustration summarizing the distribution of progenitor and neuronal regions on coronal sections of a 2w and 6w old telencephalon at mid-anterior-posterior level. (C) The fraction (%) of the total hemispheric surface covered by SV2 immunohistochemical signal was measured on coronal sections, taken at the three predefined anterior-posterior levels - anterior, mid, and posterior (suppl fig. 3B)- of 5dph, 2w and 6w old telencephalons. Quantification was performed for individual hemispheres from 4-8 fish per time point (5dph: n=24, n=24, n=21; 2w: n=8, n=13, n=16; 6w: n=16, n=15, n=20 for anterior, mid, and posterior levels, respectively). Statistical analysis was conducted using a two-way ANOVA followed by post-hoc multiple comparisons against 5dph. Significant differences are denoted with an asterisk (*$p < 0.05$, **$p < 0.01$, ***$p < 0.001$, ****$p < 0.0001$). **(D-F)** Immunohistochemical staining for SV2 (magenta) in combination with a nuclear stain (DAPI, blue) on coronal sections at the mid-anterior-posterior level at three stages of development. Scalebars: **(D)** 50 µm, **(B,E)** 100 µm, **(A,F)** 200 µm.

A

Age	Subpallium (D1) (µm)	Left hemisphere (D2) (µm)	Right hemisphere (D2) (µm)	Pallium 1 (D3) (µm)	Pallium 2 (D4) (µm)	Left hemisphere (D5) (µm)	Right hemispher (D5) (µm)
5 dph	133,541	144,251	154,036	115,713	116,533	175,779	165,62
*5 dph	163,552	169,456	170,127	128,026	125,739	210,858	213,64
5 dph	128,104	169,486	174,447	96,001	63,669	164,722	194,01
2w	254,830	254,111	284,127	209,637	246,744	356,258	354,20
2w	274,402	288,720	280,538	268,670	309,524	412,853	364,98
*2w	284,806	242,991	316,736	225,995	263,182	451,893	438,17
*6w	538,279	444,316	386,194	560,751	810,033	824,340	786,91
6w	565,421	482,127	479,608	650,389	876,495	886,433	901,46
6w	495,437	345,477	363,471	449,008	635,679	781,648	797,05

B

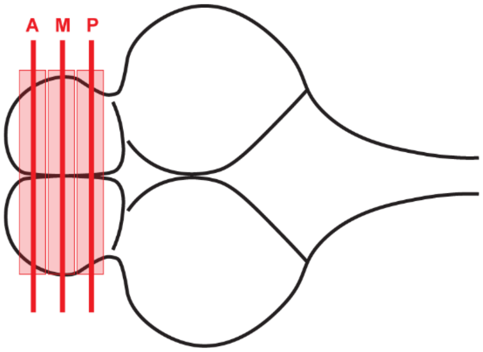

C

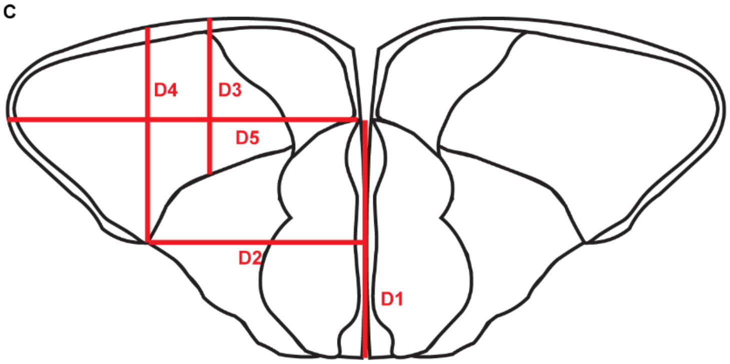

Fig. S3. Measurements of the pallial and subpallial expansion (A) Table with distances (D) measured from sections of 5dph, 2w and 6w old fish (n=3). The left and right hemispheres of the same section are separately measured for D2 and D5 (n=6). Because of the cutting angle, these measurements may vary slightly. The sections depicted in Fig. 1 and Suppl. Fig. 2. are indicated with an asterisk. **(B)** The anterior (A), mid (M) and posterior (P) levels of the telencephalon are indicated with a red box. For all measurements in A, sections of the same mid-anterior-posterior level were selected. **(C)** Illustration of all distances measured in A on a coronal section of a 2w old juvenile telencephalon.

Biology Open • Supplementary information

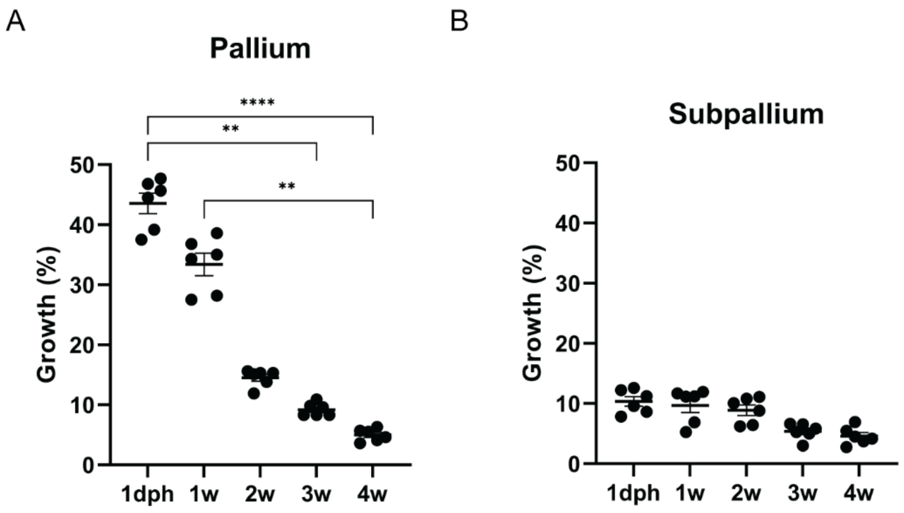

Fig. S4. Difference in pallial and subpallial growth during early life. The percentage of (A) pallial and (B) subpallial surface formed from 1dph, 2w, 3w, and 4w to 6 weeks was measured relative to the total surface area present at 6w. Growth from each time point was calculated based on the EdU labeling pattern traced until 6w. Quantification was performed on six individual hemispheres (n=6) from a minimum of three fish per timepoint, using sections from the mid-anterior-posterior level. Statistical analysis was conducted using a Kruskal-Wallis test with post-hoc multiple comparisons against 1dph. Significant differences are denoted with an asterisk (*p < 0.05, **p < 0.01, ***p < 0.001, ****p < 0.0001).

Biology Open • Supplementary information

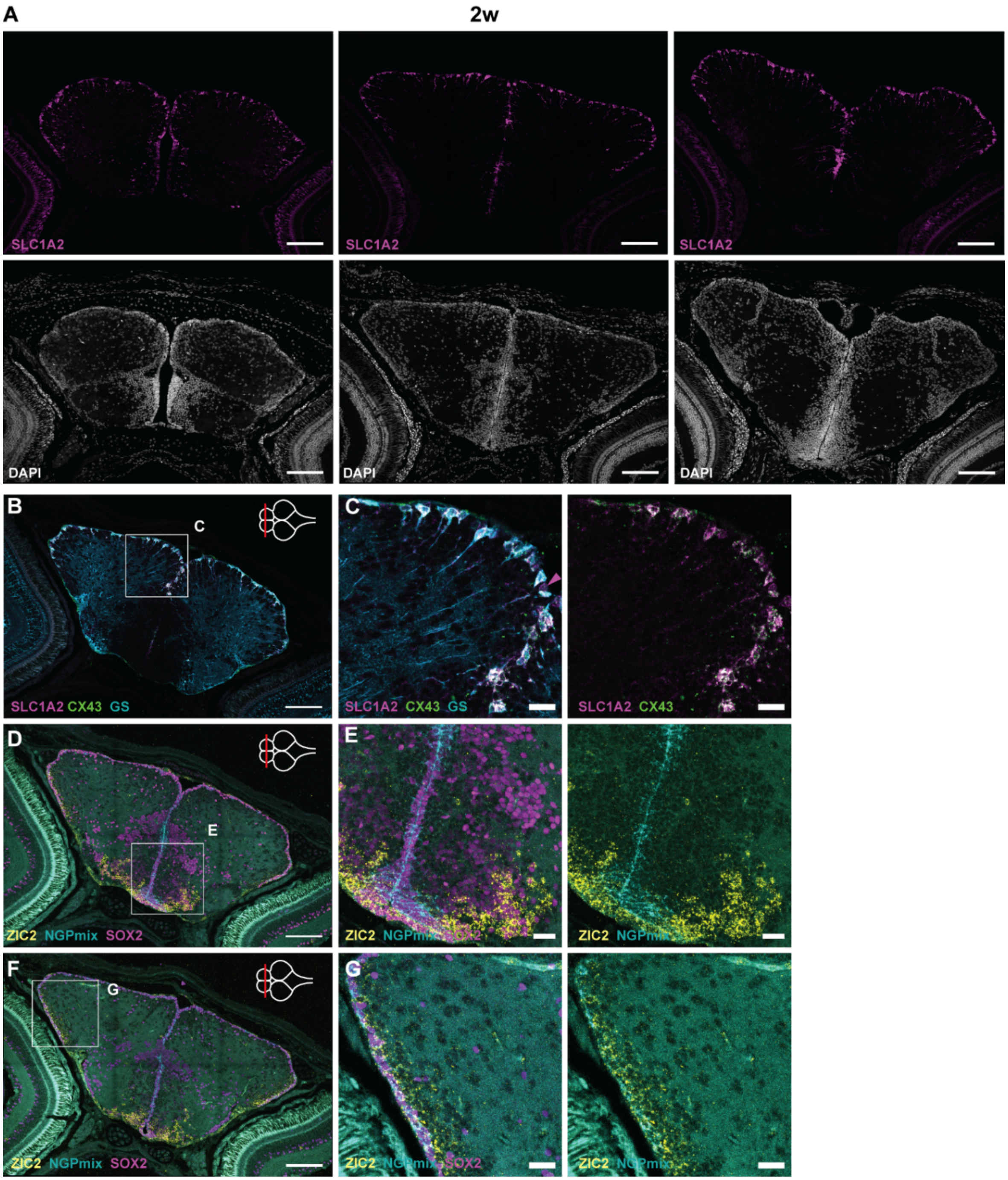

Fig. S5. Progenitors in the developing telencephalon. (A) Coronal sections of the telencephalon at 2w along the anterior-posterior axis. The top panels show an HCR-FISH targeting SLC1A2 (magenta). Cell bodies and fiber structures are visible on all levels. In the bottom panels, the nuclear composition and density of the 2w telencephalic domains is visualized with the nuclear stain DAPI (white). **(B)** HCR-FISH targeting SLC1A2 (magenta) and CX43 (green) in combination with the immunohistochemical GS (turquoise) staining on a coronal section at the mid-anterior-posterior level of the 2w telencephalon. **(C)** Magnification of the square in panel B. SLC1A2$^+$ RGs (magenta arrowhead) can be found in between clusters of mature GS$^+$/SLC1A2$^+$/CX43$^+$ RGs. **(D)** HCR-FISH targeting NGPmix (=STMN1A + HMGB2A, NGPs, turquoise) and ZIC2 (NE-RGs, yellow) in combination with an immunohistochemical staining for SOX2 (magenta). **(E)** Magnification of the square in D. The distribution of the NGPs and NE-RGs in the subpallium at 2w. **(F)** HCR-FISH targeting NGPmix (=STMN1A + HMGB2A, NGPs, turquoise) and ZIC2 (NE-RGs, yellow) in combination with an immunohistochemical staining for SOX2 (magenta). **(G)** Magnification of the square in F. The distribution of the NGPs and NE-RGs in the pallium at 2w. **(A,B,D,F)** Scale bar: 100 µm. **(C,E,G)** Scale bar: 20 µm.

Biology Open • Supplementary information

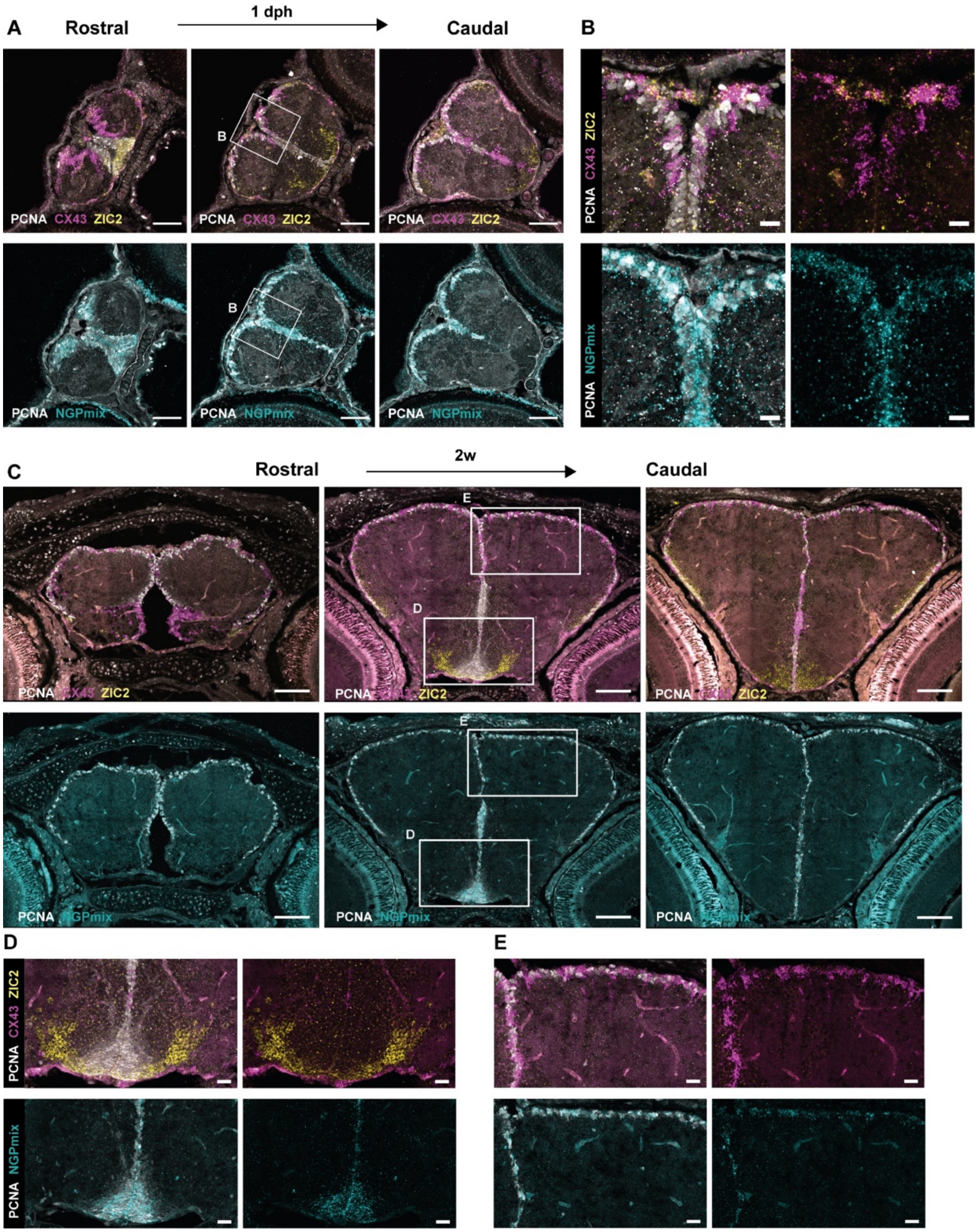

Fig. S6. Progenitor heterogeneity in the developing telencephalon. (A) Adjacent coronal sections of the telencephalon at 1dph along the anterior-posterior axis. HCR-FISH targeting (top) CX43 (pan Astro-RG, magenta) and ZIC2 (NE-RG, yellow) or (bottom) NGPmix (turquoise), is combined with an immunohistochemical staining for the proliferation marker PCNA (white). Scale bar: 50 µm. **(B)** Magnification of the squares in A. Scale bar: 10 µm. NGPs are highly proliferative at 1dph. NE-RGs, prominent at the midline, are proliferative at the posterior ventricular surface and midline. **(C)** Adjacent coronal sections of the telencephalon at 2w along the anterior-posterior axis. HCR-FISH targeting (top) CX43 (Astro-RG, magenta) and ZIC2 (NE-RG, yellow) or (bottom) NGPmix (turquoise), is combined with an immunohistochemical staining for the proliferation marker PCNA (white). Scale bar: 100 µm. telencephalon **(D-E)** Magnification of the squares in C. NGPs account for the bulk of proliferation, NE-RGs appear non-dividing in the parenchyma and dividing at the ventricular midline and posterior pallium. A general decrease in proliferating Astro-RGs is observed at 2w. Scale bar: 20 µm.

Biology Open • Supplementary information

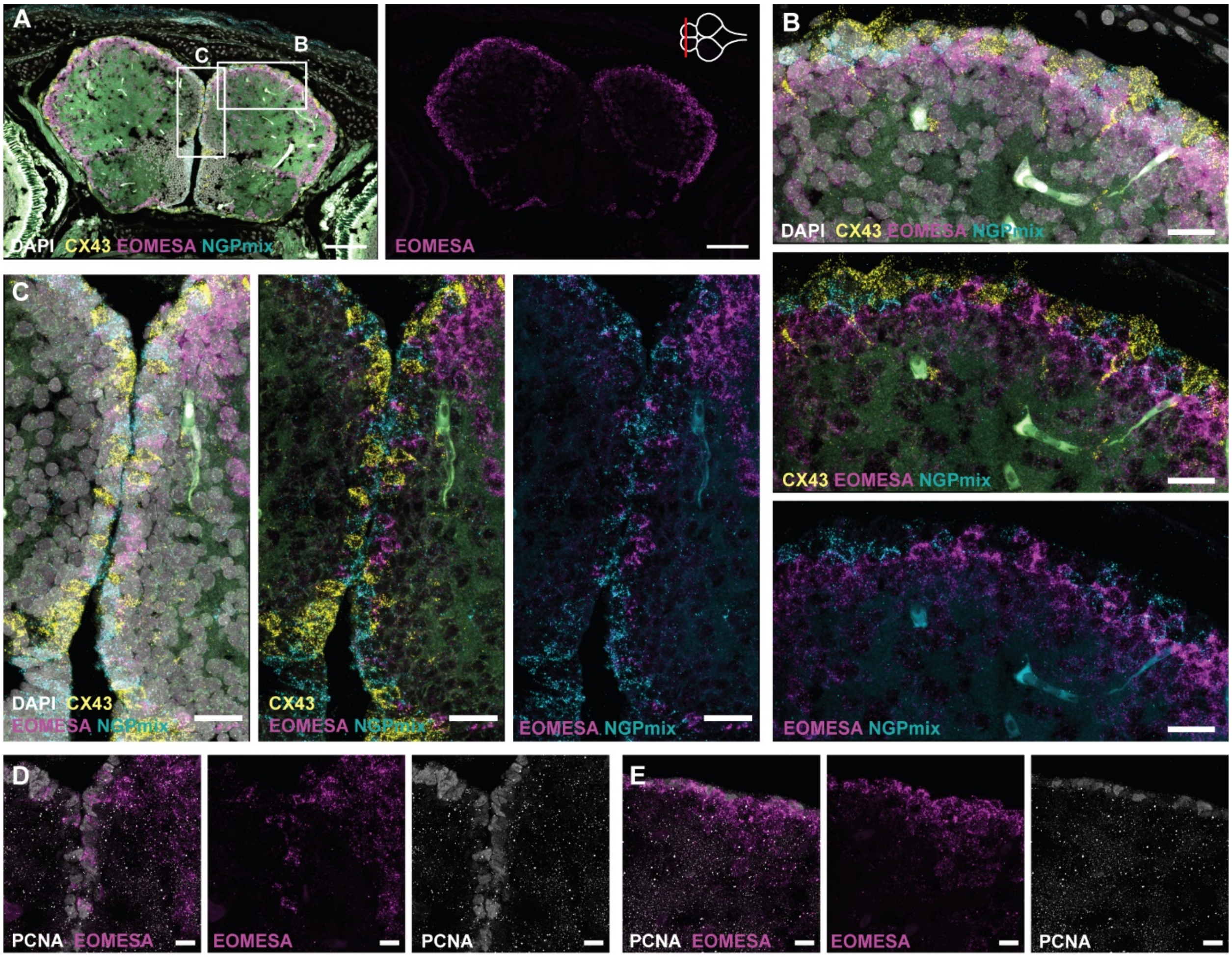

Fig. S7. Intermediate progenitors in the maturing killifish pallium (A) HCR-FISH targeting CX43 (Astro-RGs, yellow), NGPmix (turquoise) and the immature excitatory neuron marker EOMESA (magenta) in combination with the nuclear stain DAPI (white) on a coronal section of the telencephalon at 2w. The anterior-posterior level is depicted with a red line on the brain top view illustration in the top right corner. Intermediate progenitors (EOMESA^{+}/NGPmix^{+}) are located in between Astro-RGs and NGPs at the pallial ventricular surface. Scale bar: 100 µm. **(B-C)** Magnification of the squares in A. Scale bar: 20 µm. **(D-E)** HCR-FISH targeting EOMESA (magenta) in combination with an immunohistochemical staining for the proliferation marker PCNA (white). The panels are magnifications of zones on a section of comparable anterior-posterior level as in A. D and E are magnifications of the dorsal midline and pallial surface, respectively. PCNA^{+}/EOMESA^{+} cells are present at the pallial midline and surface. Scale bar: 10 µm.

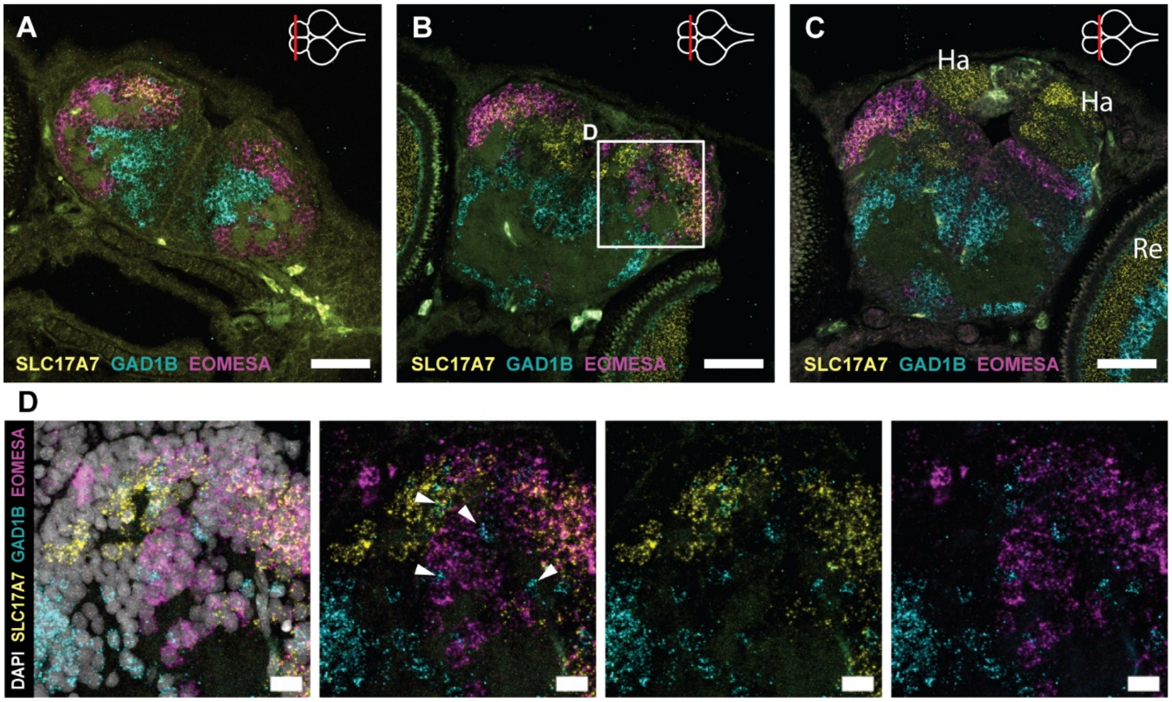

Fig. S8. Excitatory and inhibitory neuronal clusters in the 1dph telencephalon. (A-C) Coronal sections of the telencephalon at 5dph along the anterior-posterior axis. HCR-FISH targeting EOMESA (magenta) and SLC17A7 (yellow) mRNA expression shows the distribution of immature and mature excitatory neurons, respectively. This is combined with GAD1B (turquoise) labeling, visualizing mature inhibitory neurons. The anterior-posterior position of the sections is as follows: (A) mid-anterior, (B), mid-posterior and (C) posterior-most. The border of the telencephalon (posterior-most sections) is recognizable from the presence of the habenula (Ha) on top of the dorsal pallial surface, positive for SLC17A7. In the retina (Re), layers positive for predominantly SLC17A7 or GAD1B are clearly distinguishable. **(D)** Magnification of the square in panel B. HCR-FISH labeling EOMESA, SLC17A7 and GAD1B in combination with a nuclear stain (DAPI, white). GAD1B^{+} cells are found in the pallium (white arrowheads) **(A-C)** Scale bar: 50 µm **(D)** Scale bar: 10 µm.

Table S1. List of Probe pools used for hybridization chain reaction.

Probe pool	Amplifier	Nr. of probe pairs	Probe (pmol)	Concentration
B3-CX43	B3	21	0.9	
B1-SLC1A2	B1	21	0.3	
B1-NGPmix	B1	25	1.8	
B2-ZIC2	B2	24	1.8	
B3-GAD1B	B3	23	1.8	
B1-DLX1	B1	18	1.8	
B4-Slc17a7	B4	22	1.8	
B2-EOMESA	B2	24	1.8	

Table S2. List of primary and secondary antibodies used for immunostainings.

Primary/Secondary	Antibody Name	Company	Dilution
Primary	Anti-SOX2, rabbit, SAB2701973	Sigma-Aldrich	1:1000
Primary	Anti-PCNA, rabbit, GTX124496	GeneTex	1:500
Primary	Anti-Glutamine Synthetase, mouse, 3B6	Abcam	1:1000
Primary	Anti-HuC/HuD, mouse, 16A11	Invitrogen	1:100
Primary	Anti-SV2, mouse, AB_2315387	DSHB	1:500
Secondary	Donkey anti-Rabbit IgG (H+L) Cross-Adsorbed, Alexa Fluor™ 488, A - 21206	Invitrogen	1:300
Secondary	Donkey anti-Rabbit IgG (H+L) Cross-Adsorbed, Alexa Fluor™ 647, A-31573	Invitrogen	1:300
Secondary	Donkey anti-Mouse IgG (H+L) Cross-Adsorbed, Alexa Fluor™ 488, A-21202	Invitrogen	1:300
Secondary	Donkey anti-Mouse IgG (H+L) Cross-Adsorbed, Alexa Fluor™ 555, A-31570	Invitrogen	1:300

Biology Open • Supplementary information
